# Identification and Characterization of Non-*Saccharomyces* Species Isolated from Port Wine Spontaneous Fermentations

**DOI:** 10.3390/foods9020120

**Published:** 2020-01-23

**Authors:** Denisa Mateus, Susana Sousa, Cláudia Coimbra, Frank S. Rogerson, João Simões

**Affiliations:** 1 Genomics Unit, Biocant-Biotechnology Innovation Center, Biocant Park, Núcleo 04 Lote 3, 3060-197 Cantanhede, Portugal; denisa.mateus@biocant.pt (D.M.); Susana.sousa@biocant.pt (S.S.); 2 Ângelo Coimbra, S.A., Zona Industrial Maia, Sector IV Moreira, 4470-460 Maia, Portugal; claudia@angelocoimbra.pt; 3 Symington Family Estates, Vinhos S.A. Travessa Barão de Forrester 86, Apartado 26, 4431-901 Vila Nova de Gaia, Portugal; Frank.Rogerson@Symington.com

**Keywords:** non-*Saccharomyces*, wine yeasts, Port wine, native yeasts, strain characterization

## Abstract

In winemaking, non-*Saccharomyces* yeast species contribute important organoleptic complexity. Current interest focuses on abundant and dominant strains characteristically present in the early phase of spontaneous alcoholic fermentations. Non-*Saccharomyces* species are particularly relevant in Port wine production such that the fermentation is prematurely stopped, after the metabolism of only one half of the available sugar, through fortification with aguardente. This work aimed to isolate, identify and characterize non-*Saccharomyces* species present in spontaneously fermenting Port. To accomplish these goals, yeasts were isolated from a selection of frozen must samples (2012–2016 harvests), using a pre-screening process choosing only the best candidates based on the organoleptic quality of the corresponding fortified wine. From five hundred non-*Saccharomyces* isolates, twelve species were identified. The three most abundant species, *Hanseniaspora uvarum*, *Lachancea thermotolerans*, and *Metschnikowia pulcherrima*, representing 89% of the isolates, exhibited particularly high diversity with high growth performance variability when exposed to typical stress conditions associated with common enological parameters. Less abundant species included *Issatchenkia orientalis*, *Torulaspora delbrueckii*, *Hanseniaspora vineae*, *Hanseniaspora osmophila*, *Candida zemplinina*, *Rhodotorula mucilaginosa*, *Hanseniaspora guilliermondii*, *Issatchenkia occidentalis*, and *Zygosaccharomyces bisporus*. This is the first study providing insights into the identification and characterization of non-*Saccharomyces* species responsible for spontaneous Port wine production.

## 1. Introduction

Alcoholic fermentation (AF) of grape juice into wine is a complex microbiological process involving various microorganisms, including mostly yeasts, but also filamentous fungi and lactic acid bacteria. All these microorganisms leave their footprint depending on how long they are present and their dominance during the process [1]. Depending on their role in AF, yeasts are usually divided into two categories, the *Saccharomyces* and the non-*Saccharomyces* species. The latter proliferate normally at the beginning of AF, due to their lower tolerance to ethanol, and are then gradually replaced by *Saccharomyces cerevisiae*, which resists higher ethanol concentrations and has a faster growth rate [2,3]. Non-*Saccharomyces* species originate mainly from the vineyard environment, including the soil and surface of the vines and grapes being conditioned by external factors, such as grape variety, geographical location, climatic conditions, and viticulture practices, including grape maturity, vineyard spraying, pesticide treatment, and development of fungal plagues [4,5,6,7]. To a lesser extent, non-*Saccharomyces* species may also originate from the winery environment, including air, floor, and winery equipment [8,9,10]. 

In the 80s, non-*Saccharomyces* species were associated with wine spoilage and the production of undesirable compounds, such as acetic acid, ethyl acetate, and acetaldehyde [11,12]. To reduce the risk of spoilage of the wine, producers started to use commercial *Saccharomyces* yeast strains, which accelerated and standardized the AF [10,13,14], although this contributed to reducing indigenous strain diversity and the loss of wine aromatic complexity [15,16]. Nowadays, non-*Saccharomyces* species are associated with higher aromatic complexity and positive organoleptic characteristics of wines, often absent in *S. cerevisiae* [3,9]. The positive characteristics standing out include the production of aroma related compounds, such as esters, higher alcohols, and acids; the capacity to secrete enzymes that enrich wine aroma, namely esterases, β-glycosidases, lipases, proteases, and others [16,17,18]; the bioprotective effect by inhibiting spoilage microorganisms [11]; and the capacity to ferment wines with a lower alcohol content [19]. Currently, the use of native non-*Saccharomyces* species as starters is an approach of growing importance [20,21,22,23,24,25,26]; however, it is necessary to isolate, identify, and characterize the native strains.

Port wine is a fortified wine produced in the world’s oldest demarcated and regulated winemaking region, the Douro Demarcated Region (DDR), located in Northeast Portugal. This is considered the most arid winemaking region in Europe due to its characteristic hot dry summers, and its schistous soil with acidic pH, rich in potassium and poor in organic matter, phosphorus, magnesium, and calcium [27,28]. The vineyards planted on steep slopes protected by mountains surrounding the Douro river benefit from a diversity of microclimates [27,28]. Port wine is normally produced by spontaneous AF, mainly assured by native non-*Saccharomyces* species, which is prematurely interrupted with the addition of a distilled grape spirit (known as “aguardente”) [29], [30]. Port wine stands out from common wines due to its particular characteristics, including the wide variety of styles, according to alcohol content (usually between 19% and 22% vol., excluding the white semi-dry that must have a minimum of 16.5% vol.), sweetness (from <40 g/L sugar content for the semidry, to >130 g/L sugar content for the very sweet wines), and assortment of colors (red ports vary from deep purple to light gold, and white ports from pale yellow, straw, and golden white) [27,29]. 

Nowadays winemakers are concerned about producing not only high-quality wines but also wines that link sensory features to the terroir, which is influenced by (1) physical factors, such as the climate; (2) biological factors, including soil, grape variety, and fauna; (3) human-agronomical practices in the vineyards and in the cellar; and (4) the native vine microbiota [31]. To respond to these challenges there is an increasing interest to study and identify the native yeast population, with an emphasis on non-*Saccharomyces* species colonizing grapes and vineyards. To our knowledge, there are no studies on the yeast population, namely of non-*Saccharomyces* species, present in Port wine AF. The aim of this work was to isolate, identify, and characterize native non-*Saccharomyces* species present in spontaneously fermented Port wine musts. Isolated non-*Saccharomyces* species were identified at the strain level before being submitted to technological characterization, where growth performance in stress conditions typically associated with AF and related oenological parameters were evaluated. 

## 2. Materials and Methods 

### 2.1. Sample Collection and Yeast Isolation

A total of 22 cryopreserved Port wine must samples were pre-selected for the corresponding superior quality of the fine Port wines (*n* = 13 wines selected for the study), produced during 5 consecutive harvesting campaigns (2012 to 2016). Details of the samples are in Supplementary Material (Table S1). Monovarietal Port wines were fermented using the top two single red grape varieties, Touriga Nacional (*Vitis vinifera L*.), known to produce the highest-quality Port wines, and Touriga Franca (*V. vinifera L*.), the most popular variety for Port wine production [27,29]. Grapes were harvested from vineyards of the Symington Family Estates group, located in the Douro Demarcated Region of Portugal. Must samples were collected in situ, at two stages of the micro-scale spontaneous AF, and cryopreserved at −50 °C in sterile tubes containing 30% glycerol [32,33]. Following sensory evaluation of the corresponding fermented Port wines, yeasts were isolated from only the most promising must samples, collected at the two different stages of AF: the beginning, enriched in non-*Saccharomyces* yeasts [34], and the middle stage, prior to fortification, indicative of dominant strains. Serial ten-fold dilutions (10^−2^ to 10^−6^) of cryopreserved must samples were plated on selective lysine agar medium (Oxoid) [35] and on YEPD agar medium (1% yeast extract, 2% peptone, 2% glucose, and 2% agar). After incubation at 25 °C, for 3–5 days, up to 20–30 colonies were isolated from dilution plates containing ~30–300 colonies. For plates with >300 colonies, unique colonies having different morphological phenotypes were also characterized [36].

### 2.2. Identification of Yeast Species

From all yeast isolates the genomic DNA was extracted with the Wizard Genomic DNA Purification Kit (Promega), quantified and frozen at −20 °C until later use. Yeast isolates were identified by PCR analysis of the internal transcribed spacer region (ITS), which comprise the 5.8S rRNA and two flanking regions (ITS1 and ITS2), with the oligonucleotides ITS1 and ITS4 [37]. PCR products were analyzed by electrophoresis on 1.5% agarose gel. Results obtained with the ITS-PCR analysis were confirmed by Sanger-sequencing of the ITS region. For this, the ITS-PCR product was first purified with an Illustra ExoProStar kit (GE Healthcare Life Sciences, Pittsburgh, EUA), and sequencing reactions were prepared with the BigDye™ Terminator v3.1 Cycle Sequencing Kit (Applied Biosystems, Foster City, Califórnia, EUA). Sequencing reactions were purified with BigDye XTerminator (Applied Biosystems, Foster City, Califórnia, EUA) and analyzed in the 3500 Genetic Analyzer (Applied Biosystems, Foster City, Califórnia, EUA). Sequence reactions were analyzed with BioEdit software and with the Basic Local Alignment Search Tool (BLASTn-NIH). From a total of 568 isolates, 500 were identified as non-*Saccharomyces* species.

### 2.3. Clonal Characterization of Non-Saccharomyces Species

Clonal characterization of selected non-*Saccharomyces* isolates from the three most represented species, *Hanseniaspora uvarum* (123/277 isolates), *Lanchacea thermotolerans* (40/85 isolates), and *Metschnikowia pulcherrima* (39/83 isolates) was performed by RAPD-PCR analysis with the oligonucleotides M14 (5′-GAGGGTGGCGGTTCT-3′) [38] and P80 (5-CGCGTGCCCA-3) [39], as previously described with slight modifications. The PCR reactions were performed in 15 µL of a mix containing 50 ng of DNA template, 1X incomplete reaction buffer (Bioron, Römerberg, Germany), 3 mM of MgCl_2_, 0.2 mM dNTPs mixture, 1 µM of oligonucleotides, and 0.04U of Taq DNA polymerase (Bioron, Römerberg, Germany). The PCR program was 94 °C for 5 min, 40 cycles at 94 °C for 1 min, Tm (for P80 Tm = 36 °C, for M14 Tm = 45 °C) for 1 min, 72 °C for 1 min, plus a final extension at 72 °C for 5 min. PCR products were analyzed on 1.5% agarose gels in 1X Tris–borate EDTA, at 100 V for 2 h, and the size of the DNA fragments was estimated by comparison with the DNA molecular weight marker-NZY DNA ladder I (NZYTech´, Lisboa, Portugal). Gel images were captured with Gel doc XR (BioRad, Hercules, Califórnia, EUA) equipped with the software Image Lab version 5.1 (BioRad, Hercules, Califórnia, EUA). RAPD-PCR profiles obtained were analyzed using GelJ v 2.0 software [40]. Dendrograms obtained by hierarchical clustering of the band fingerprints were generated using the Dice similarity coefficient, with a tolerance of 10 and the UPGMA algorithm. Samples were considered to be of the same strain within a species when similarity values were ≥80%.

### 2.4. Phenotypic Screening of Selected Non-Saccharomyces Strains

Phenotypic screening enabled the evaluation of the growth performance of non-*Saccharomyces* species during applied stress conditions associated with AF. Isolates were selected to include at least one representative of each strain group identified previously by RAPD-PCR. Two commercial non-*Saccharomyces* strains, *L. thermotolerans* (VINIFLORA^®^ CONCERTO, CHR Hansen, Hørsholm, Dinamarca) and *Torulaspora delbrueckii* (VINIFLORA^®^ PRELUDE, CHR Hansen, Hørsholm, Dinamarca), and the commercial *S. cerevisiae* (VINIFLORA^®^ MERIT, CHR Hansen, Hørsholm, Dinamarca) were included as experimental controls. Isolates were grown in YEPD medium with different concentrations of ethanol (2.5%, 5%, 7.5%, 10%, and 12.5%-the appropriate volume of ethanol absolute-99.9% *v*/*v* analytical grade was added to YEPD before plating); osmotic stress (sorbitol 20% and 30%-added to YEPD before autoclaving); acidic pH’s (3.0 and 3.4-pH of liquid YEPD was adjusted with HCL 37%); high temperature (30 °C and 37 °C); nitrogen sources (YNB + essential amino acids-L-proline, L-glutamine and L-arginine); and different carbon sources (YEP with 2% fructose, and 10% glucose + 10% fructose-sterile solution of sugars was added to autoclaved YEP). Isolates and the reference controls were first grown on 96-deep well plates containing 2 mL of YEPD for 48 h, at 25 °C, and 100 rpm. A 96-well source plate was prepared by adjusting cell densities to 1 × 10^7^–1 × 10^5^ cells/ml with sterile water. The source plate was automatically arrayed onto target agar plates with the robotic Sciclone ALH 200 Advanced Liquid Handling System, using a 96-pin tool (Caliper Life Sciences, Waltham, Massachusetts, EUA). Target plates were incubated for 48 h at 25 °C, or at 30 °C and 37 °C to evaluate the growth performance at higher temperatures. Images of plates were captured with a DSLR camera on a fixed tripod with focal length and f-stops acquisition settings identical. Images were processed using ImageJ software. The growth advantage or disadvantage of the isolates was calculated based on the variation of the colony size area quantified in the plates supplemented with stress conditions, relative to those quantified in the control plates (YEPD). Biological triplicates were performed from three independent growth experiments. Heatmaps and hierarchical clustering were constructed using MultiExperimental Viewer (MeV 4.9.0) software, applying the Pearson correlation. A principal component analysis (PCA) plot was constructed with Unscrambler multivariate analysis software by CAMO. Raw data are in Supplementary Material Table S2.

### 2.5. Metabolite Characterization of Selected Non-Saccharomyces Strains

Selected non-*Saccharomyces* strains, performing better in the phenotypic screening, including 17 *H. uvarum*, 6 *L. thermotolerans*, 5 *M. pulcherrima,* and 1 *Issatchenkia orientalis*, were inoculated, in triplicates, at an initial optical density of 0.1 on 96-deep well plates containing 1 mL of synthetic must [41] (with slight modifications, including yeast extract 5 g/L, malic acid 3 g/L, citric acid 0.7 g/L, tartaric acid 2 g/L, KH_2_PO_4_ 1.14 g/L, MgSO_4_.7H_2_O 1.23 g/L, CaCl_2_.3H_2_O 0.44 g/L, MnSO_4_.H_2_O 170 mg/L, ZnSO_4_.7H_2_O 285 mg/L, CuSO_4_.5H_2_O 28 mg/L, Co(NO_3_)_2_.H_2_O 37 mg/L, and FeSO_4_.7H_2_O 66 mg/L, with a final pH of 3.3). Isolates were grown at 30 °C, at 240 rpm for 72 h. Six 96-deep well plates were inoculated simultaneously for analysis for the selected 6-time points (2 h, 22 h, 30 h, 46 h, 54 h, and 72 h after incubation). For each time point one of the plates for measurement was selected. An optical density at 600 nm (OD_600_) was measured in the spectrophotometer for each of the selected time points. Concentration levels of ethanol, glucose, fructose, glycerol, and mannitol were quantified by high-performance liquid chromatography (HPLC), Shimadzu-LC-2010C HT, equipped with a refractive index detector and using a Rezex 8 μm RPM Monosaccharide Pb^+2^ (8%) LC 300 × 7.8 mm column, with MilliQ water as the mobile phase, at a flow rate of 0.6 ml/min, for each of the selected time points. The concentration of organic acids (tartaric, malic, succinic, pyruvic, citric, acetic, and lactic acid) was quantified by HPLC, Shimadzu-LC-20AD, equipped with a refractive index detector and an ultraviolet detector at 210 nm, using a Shodex Sugar SH1011, 8.0 mm ID × 300 mm column, with 5 mM sulfuric acid as the mobile phase, at a flow rate of 0.6 mL/min, for each of the selected time points. The concentration of each metabolite was calculated using external standards. Samples were analyzed in triplicate, corresponding to three independent growths. Graphics were generated using the GraphPad Prism 4 software. The principal component analysis (PCA) plot was constructed with Unscrambler multivariate analysis software by CAMO. Raw data are in Supplementary Material Table S2.

## 3. Results

### 3.1. Identification and Clonal Characterization of Non-Saccharomyces Species Isolated from Spontaneously Fermented Port Wine Musts

**Figure 1:**
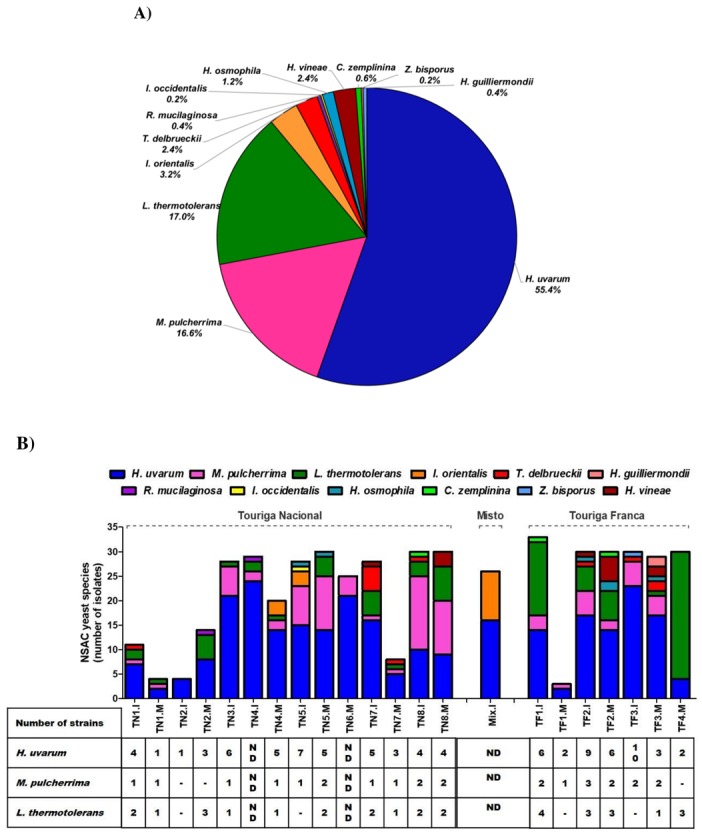
Distribution of non-*Saccharomyces* species isolated from Port wine must samples. (**A**) The total distribution of non-*Saccharomyces* species isolated from Port wine must samples. Port wine must samples were plated in YEPD and lysine medium, and the identification of the yeast isolates was performed by PCR analysis of the 5.8-ITS rRNA region. (**B**) Strain distribution in Port wine must samples collected at the beginning of AF (“I” samples) compared to a later stage, prior to fortification (“M” samples). The number of strain clusters for *H. uvarum*, *L. thermotolerans*, and *M. pulcherrima* species in each must sample is represented in the table. Strains were analyzed by RAPD-PCR and grouped in the same cluster when similarity values were ≥80% (Dice similarity coefficient with a tolerance of 10, and the UPGMA algorithm).

From a total of 568 yeasts isolated from spontaneously fermented Port wine musts, 500 non-*Saccharomyces* yeasts from twelve species were identified. *H. uvarum* was the most dominant species, corresponding to 55% of all the isolates, followed by *L. thermotolerans* and *M. pulcherrima*, which represented, respectively, 17% and 16.6%. The other non-*Saccharomyces* species, identified in a lower frequency (<4%), included *I. orientalis*, *T. delbrueckii*, *H. vineae*, *H. osmophila*, *Candida zemplinina (Starmerella bacillaris)*, *Rhodotorula mucilaginosa*, *H. guilliermondii*, *I. occidentalis,* and *Zygosaccharomyces bisporus* (Figure 1A).

Clonal characterization was performed by RAPD-PCR analysis for the three dominant species, respectively, *H. uvarum*, *L. thermotolerans*, and *M. pulcherrima*. The RAPD-PCR enabled strain differentiation within species, according to the PCR patterns amplified randomly with specific oligonucleotides (representative image in Supplementary Figure S1). The oligonucleotide P80 was used to differentiate *H. uvarum* strains [39], whilst M14 was used to differentiate *L. thermotolerans*, and *M. pulcherrima* strains [38]. Due to the high number of *H. uvarum* isolates identified in Port wine must samples (277 isolates), the RAPD-PCR profiles obtained for this species were analyzed for each grape variety. *H. uvarum* strains isolated from the Touriga Franca grape variety were divided into 17 sub-groups of strains (Supplementary Figure S2), whilst Touriga Nacional isolates were divided into 20 sub-groups (Supplementary Figure S3). *L. thermotolerans* and *M. pulcherrima* isolates from both grape varieties were, respectively, divided into 11 and 18 sub-groups of strains (Supplementary Figures S4 and S5). Considerable strain variability was observed for *H. uvarum*, *M. pulcherrima*, and *L. thermotolerans* (Figure 1B), and was particularly high at the beginning of AF (“I” samples, collected at the beginning of AF) compared to a later stage, prior to fortification (“M” samples) (Figure 1B).

### 3.2. Phenotypic Screening of Selected Non-Saccharomyces Strains

**Figure 2:**
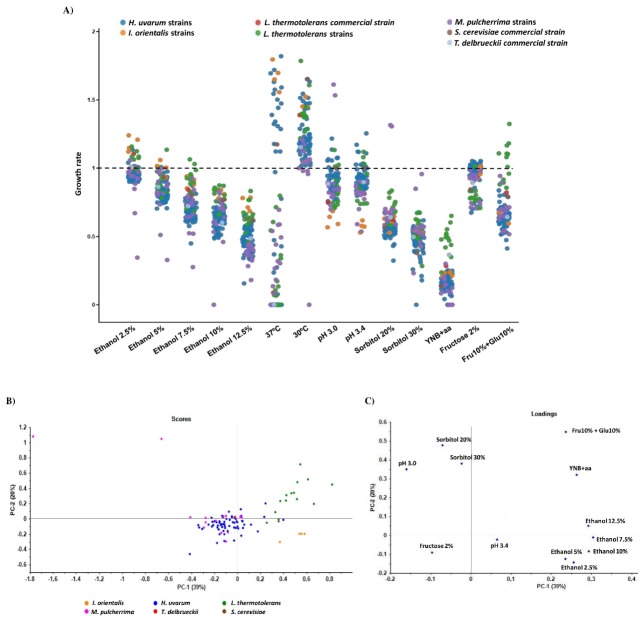
Phenotypic screening of selected non-*Saccharomyces* species isolated from Port wine must samples on stress conditions associated with alcoholic fermentation. (**A**) Growth performance of selected non-*Saccharomyces* strains on YEPD culture plates supplemented with stress conditions associated with alcoholic fermentation. Stress conditions include different concentrations of ethanol, temperatures (37 °C and 30 °C), acidic pH (3.0 and 3.4), osmotic stress (Sorbitol 20% and 30%), different nitrogen source (YNB + essential amino acids), and different carbon sources (Fructose 2% and Fructose 10% + Glucose 10%). For the assay 92 non-*Saccharomyces* isolated strains were selected, and as reference controls two commercial non-*Saccharomyces*, respectively, *L. thermotolerans* and *T. delbrueckii*, and the commercial *S. cerevisiae*. Results represent the mean value of the colony size area quantified in the plates supplemented with stress conditions, relative to that quantified in the control plates (no stress). Mean values were obtained for biological triplicates from three independent growth experiments. The PCA score plot (**B**) and loading plot (**C**) in terms of Principal Component 1 (PC 1, x-axis) and Principal Component 2 (PC 2, y-axis) for the selected *I. orientalis*, *H. uvarum*, *L. thermotolerans*, *M. pulcherrima*, and the commercial strains. Singular Value Decomposition (SVD) with imputation was used to calculate principal components.

During alcoholic fermentation yeasts are simultaneously and sequentially affected by various stressors, including osmotic pressure, acidic pH, sulfur dioxide concentration, falling oxygen levels, nutrient limitation, including nitrogen, carbon, and vitamins, high must temperatures, as well as increasing ethanol concentration, which inhibit yeast growth, leading to the finalization of the process (reviewed in [7]). Phenotypic screening was designed to evaluate the capacity of selected non-*Saccharomyces* strains to grow under stress conditions associated with AF. Representative strains, identified by RAPD-PCR, were selected giving preference to the isolates from must samples of the 2016 harvest campaign, as this was considered an exceptional quality year for Port wine. Selected strains included 61 isolates of *H. uvarum*, 13 of *L. thermotolerans*, 14 of *M. pulcherrima*, and 4 of *I. orientalis*. The *I. orientalis* isolates were included in the assay because this was the 4th most frequent species identified and presented high representativeness in one must sample (Mix.I). Two commercial non-*Saccharomyces* strains, *L. thermotolerans* and *T. delbrueckii*, and a commercial *S. cerevisiae* were included in the study for experimental control. The capacity of the isolates to grow on YEPD plates supplemented with stress factors associated with AF, including temperature (30 °C and 37 °C); nitrogen source (YNB + essential amino acids-L-proline, L-glutamine and L-arginine); carbon source (2% fructose, 10% glucose + 10% fructose); ethanol concentration (2.5%, 5%, 7.5%, 10% and 12.5%); osmotic stress (sorbitol 20% and 30%); and acidic pH (3.0 and 3.4), was quantified relatively to the growth on YEPD control plates. The growth rate of the selected isolates for the aforementioned stress conditions (Figure 2A, and Supplementary Figure S6A,B), demonstrated high variability for the selected non-*Saccharomyces* strains. The principal component analysis (PCA) plot for scores (Figure 2B), constructed with the growth rate results presented in Figure 2A, evidenced the higher heterogeneity for *L. thermotolerans* strains. The PCA plot for the loadings (Figure 2C) evidenced that stress conditions characterizing this dispersion were temperature (30 °C), yeast nitrogen medium supplemented with three essential amino acids (L-proline, L-glutamine, and L-arginine-YNB + aa) and a medium with both glucose and fructose with a final 20% concentration (Fru 10 + Glu 10%).

Applied temperature stress at 37 °C was the condition in which the greatest variance was observed (Figure 2A), compromising the growth of most *M. pulcherrima* and *L. thermotolerans* strains whilst enhancing *I. orientalis* strains. *H. uvarum* strains presented a heterogeneous response, with some isolates demonstrating a growth advantage and others a disadvantage. At 30 °C no negative growth impact was observed, with the exception of one *M. pulcherrima* isolate. The *S. cerevisiae* control and two *L. thermotolerans* strains were the isolates with the greatest growth advantage at this temperature (Figure 2A). The PCA plots (Figure 2B,C) were generated excluding growth results at 37 °C, as this extreme temperature induced the greatest variability for the selected strains, contributing excessive strain dispersion and hiding important correlation patterns. The negative growth impact observed at 37 °C only confirms the importance to regulate maximum fermentation temperatures to 30 °C.

Ethanol inhibited the growth of the selected isolates in a dose-dependent manner, with *M. pulcherrima* and *H. uvarum* strains the most sensitive, contrasting the greater tolerance of *I. orientalis* and *L. thermotolerans*, as evidenced in Figure 2A,B.

Figure 2A and the PCA plots (Figure 2B,C) evidenced the impact of the two acidic pH 3.0 and 3.4 conditions, compared with the non-modified YEPD control medium with a pH of 6.5, resulting in the slightly reduced growth of most strains, being particularly evident for *I. orientalis*.

The capacity to grow in hyperosmotic conditions was evaluated in the presence of 20% and 30% sorbitol, a non-assimilable carbon source. All isolates, with the exception of two *M. pulcherrima* strains, were sensitive to osmotic stress (Figure 2A,B). Likewise, the growth of all isolates was severely impaired in culture medium containing yeast nitrogen base (YNB) supplemented with three essential amino acids (L-proline, L-glutamine, and L-arginine) relative to the growth in the YEPD control medium (Figure 2A–C). Growth of *L. thermotolerans* strains in culture medium supplemented with 2% fructose was slightly reduced, while in medium supplemented with 10% concentrations of both glucose and fructose, it was unaffected. For all strains from other species, growth was slightly impaired in the presence of 10% glucose + 10% fructose (Figure 2A–C).

PCA plots (Figure 2B,C) evidenced the higher heterogeneity for selected *M. pulcherrima*, with two outlier strains located on the extreme of the plot. The *M. pulcherrima* strain outliers were characterized with favorable growth at both acidic pH 3.0 and higher sorbitol concentrations. On the contrary *H. uvarum* strains, despite their high clonal diversity, exhibited a more similar phenotypic response. The *I. orientalis* selected strains presented a low dispersion, consistent with high phenotypic similarity observed for this species. The isolated *L. thermotolerans* strains indicate the most similar characteristics to both *L. thermotolerans* and *S. cerevisiae* commercial strains. As expected, *L. thermotolerans* and *S. cerevisiae* commercial strains have a higher tolerance to the ethanol concentrations and temperatures tested. *H. uvarum* and *M. pulcherrima* isolates exhibited a phenotype more similar to *T. delbrueckii* commercial strain due to its lower tolerance to ethanol and temperature.

The phenotypic screening heatmaps (Supplementary Figure S6A) demonstrate that tolerance to a particular stress condition does not imply tolerance to other stress conditions.

### 3.3. Metabolite Characterization of Selected Non-Saccharomyces Strains

**Figure 3:**
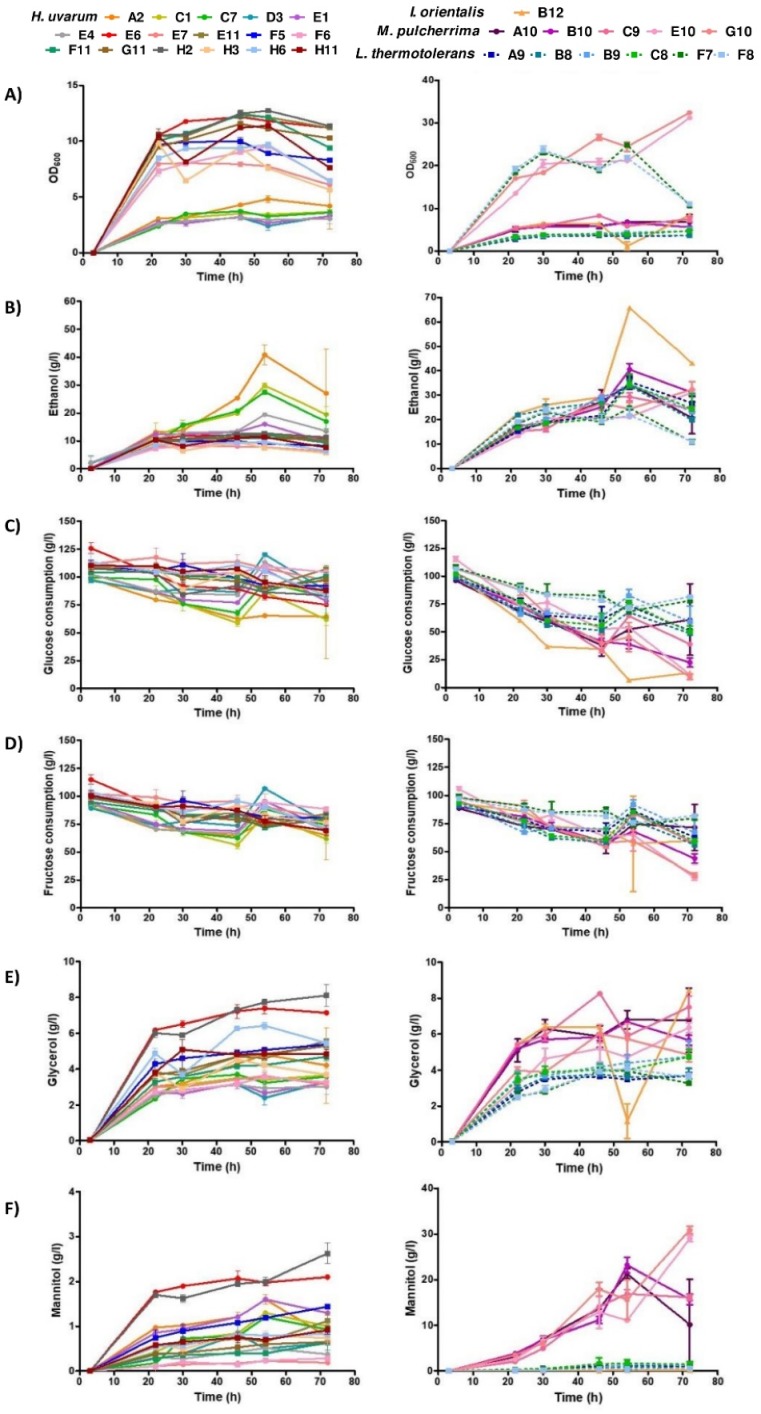
Quantification of metabolite for selected non-*Saccharomyces* species isolated from Port wine must samples. The growth (OD_600_) (**A**) was evaluated using a spectrophotometer. Ethanol (**B**) production rate, glucose (**C**), and fructose (**D**) consumption, as well as glycerol (**E**) and mannitol (**F**) concentrations were measured by HPLC. Results represent the mean value ± SD of triplicates, corresponding to three independent growths.

Selected non-*Saccharomyces* strains, including 17 *H. uvarum*, 6 *L. thermotolerans*, 5 *M. pulcherrima,* and 1 *I. orientalis*, were characterized for parameters with oenological relevance, including the quantification of glucose and fructose consumption, the ethanol production, and the concentration of glycerol, mannitol, and organic acids (tartaric, malic, succinic, pyruvic, citric, acetic, and lactic acids). At the same time points, the growth (OD_600_) of all isolates (Figure 3A) was evaluated using a spectrophotometer.

Ethanol production was considerably lower for the majority of *H. uvarum* strains compared to the other species (Figure 3B). At the exponential phase, for the studied conditions, the majority of *H. uvarum* strains produced ~1% ethanol, *M. pulcherrima* and the majority of *L. thermotolerans* strains produced 2–3%, and the *I. orientalis* B12 strain produced 4% ethanol.

Glucose and fructose consumption, which is in line with ethanol production, was higher for *I. orientalis*, following *L. thermotolerans* and *M. pulcherrima*, and lower for *H. uvarum* strains (Figure 3C,D and Figure 5A,B). Moreover, *I. orientalis* and *M. pulcherrima* strains demonstrated a clear metabolic preference for glucose instead of fructose, whilst *L. thermotolerans* strains exhibited only a slight preference for glucose. On the contrary, *H. uvarum* strains exhibited a slight preference for fructose.

Glycerol production, determined during the stationary phase (Figure 3E), was found to be considerably higher for *M. pulcherrima* and *I. orientalis* strains (~6–8 g/L) and lower for *L. thermotolerans* strains (~4 g/L). *H. uvarum* strains presented the greatest variability, with values ranging from 3–8 g/L.

The two *M. pulcherrima* strains produced the greatest levels of mannitol, reaching 30 g/L; *I. orientalis* and *L. thermotolerans* strains produced the lowest levels (Figure 3F and Figure 5A,B).

**Figure 4:**
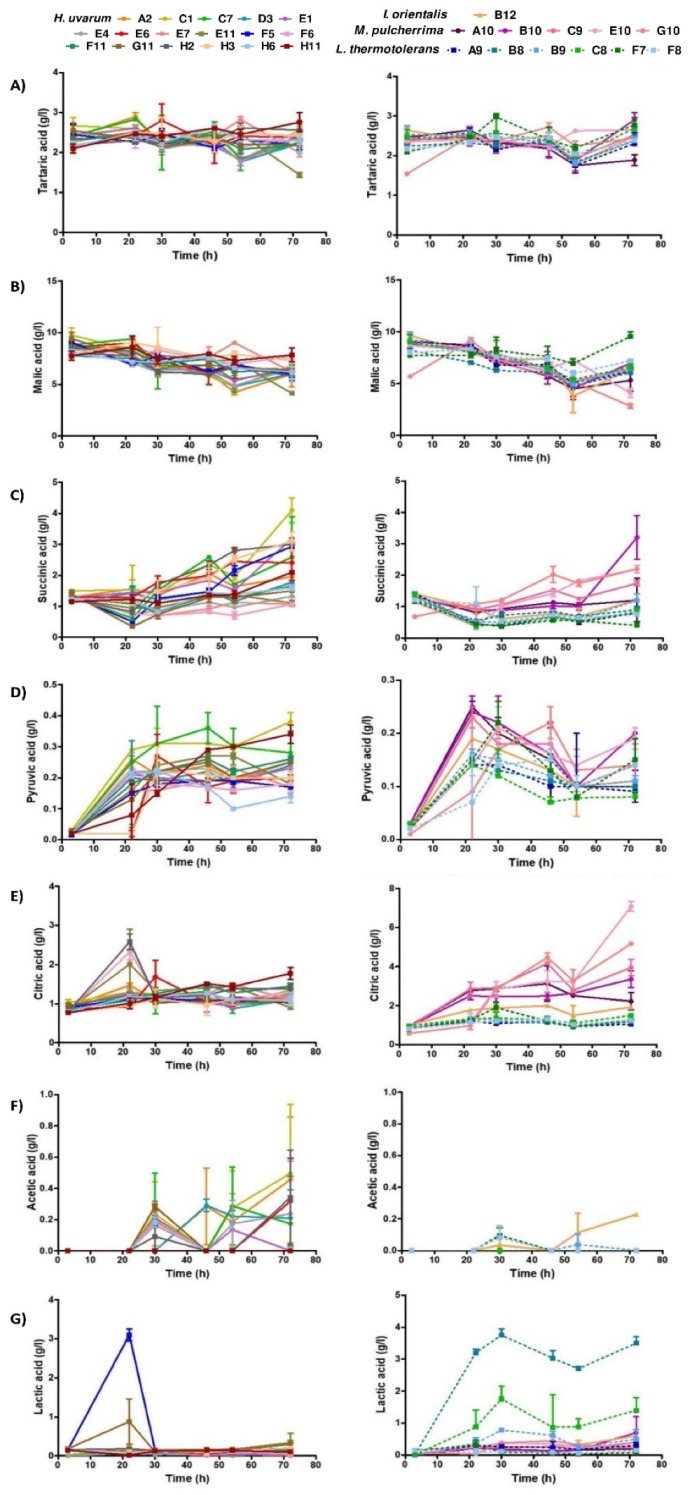
Quantification of organic acids for selected non-*Saccharomyces* species isolated from Port wine must samples. The concentration of tartaric (**A**), malic (**B**), succinic (**C**), pyruvic (**D**), citric (**E**), acetic (**F**), and lactic (**G**) acids for selected non-*Saccharomyce*s species isolated from Port wine must samples was quantified by HPLC. Results represent the mean value ± SD of triplicates, corresponding to three independent growths.

**Figure 5:**
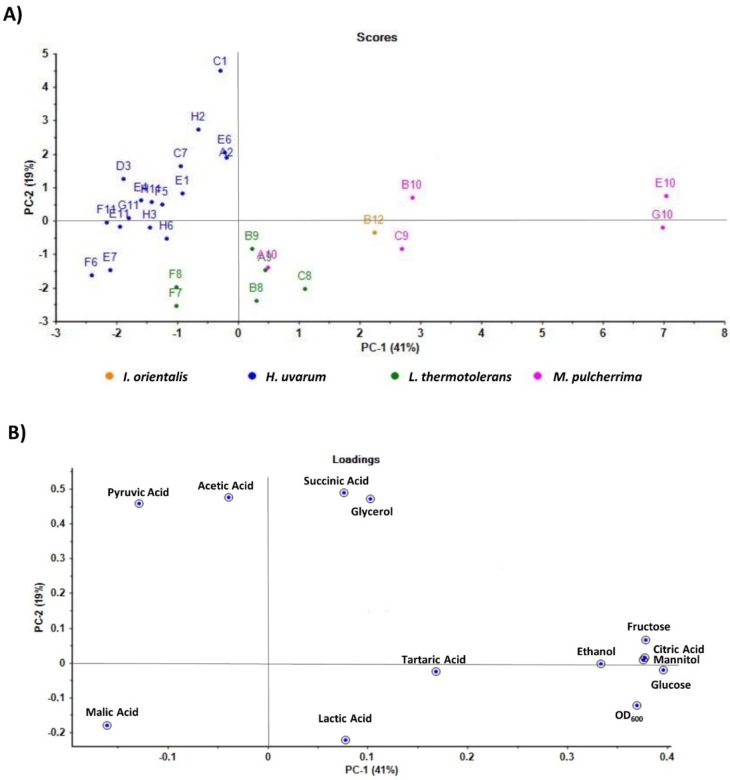
Principal component analysis (PCA) plot based on the metabolite characterization for the selected non-*Saccharomyces* species isolated from Port wine must samples. PCA score plot (**A**) and loading plot (**B**) in terms of Principal Component 1 (PC 1, x-axis) and Principal Component 2 (PC 2, y-axis) was performed with metabolite quantification results, including ethanol production rate, glucose and fructose consumption, glycerol, mannitol, and organic acids quantification, for selected non-*Saccharomyces* species isolated from Port wine must samples. Singular Value Decomposition (SVD) with imputation was used to calculate principal components.

Tartaric acid concentrations (Figure 4A), which remained constant along time, were similar for all strains/species (Figure 5A,B). In contrast, malic acid was metabolized, decreasing along time for all strains/species (Figure 4B). Succinic acid levels increased along time in the media fermented by *H. uvarum* and *M. pulcherrima* strains, but were observed to decrease fractionally between the beginning of yeast growth and the stationary phase for *I. orientalis* and *L. thermotolerans* strains (Figure 4C). Pyruvic acid, which was found present at lower relative organic acid concentrations, (<0.5 g/L), varied considerably between the selected strains (Figure 4D and Figure 5A,B). Its concentration increased considerably during the beginning of yeast growth and oscillated during the exponential growth phase, indicative of its production, followed by its consumption. Citric acid production also registered relatively low levels (<2 g/L) for *I. orientalis*, *L. thermotolerans,* and *H. uvarum* strains, whereas *M. pulcherrima* strains exhibited greater variance (2–7 g/L) (Figure 4E). For the applied experimental conditions, acetic acid production was undetectable for *I. orientalis*, *L. thermotolerans*, and *M. pulcherrima* strains (Figure 4F). Higher levels of produced acetic acid were registered for some *H. uvarum* strains, whilst others yielded minimal or undetectable levels. Final lactic acid concentrations were highest for *L. thermotolerans* strains, with highest production levels evident for the B9 strain (Figure 4G and Figure 5A,B).

Moreover, PCA plots demonstrated that *H. uvarum* and *L. thermotolerans* strains group in more compact clusters, demonstrating these strains exhibited more similar characteristics. On the contrary, *M. pulcherrima* strains group in a less compact cluster, demonstrating much higher variability for this strain.

## 4. Discussion

### 4.1. Genetic Diversity within Non-Saccharomyces Species Isolated from Spontaneously Fermented Port Wine Musts

In recent years, several studies have made major advances to characterize the native yeast population involved in wine production, mostly to understand the ecology, physiology, biochemistry and molecular biology of *Saccharomyces* and non-*Saccharomyces* species [2]. Recent studies have demonstrated the potential of non-*Saccharomyces* species to enhance wines aromatically and to increase their organoleptic complexity, mostly in mixed or sequential inoculations with *S. cerevisiae* [11,21,22,26,42,43,44,45,46,47,48,49]. Besides increasing wine organoleptic complexity, some non-*Saccharomyces* species, such as *L. thermotolerans*, *M. pulcherrima,* and *T. delbrueckii*, were previously associated with pigment stabilization through the formation of metabolic precursor compounds [50]. Thus, there is a growing interest in isolating, identifying, and characterizing the microorganisms involved in spontaneous AF, with a particular emphasis on non-*Saccharomyces* species, to explore their oenological potential. Our study aimed to isolate, identify, and characterize non-*Saccharomyces* species present in Port wine spontaneous AF. Twelve species belonging to eight genera were identified in Port wine must samples. *H. uvarum* was the predominant non-*Saccharomyces* species isolated from Port wine must samples, in agreement with previous studies performed in non-Douro vineyard regions [15,42,51,52,53,54,55]. *L. thermotolerans* and *M. pulcherrima* were identified as the second and third most predominant non-*Saccharomyces* species. The other non-*Saccharomyces* species isolated in lower concentrations included *I. orientalis*, *T. delbrueckii*, *H. vineae*, *H. osmophila*, *C. zemplinina (S. bacillaris)*, *R. mucilaginosa*, *H. guilliermondii*, *I. occidentalis*, and *Z. bisporus*. *Hanseniaspora* was the most represented genus, followed by *Lachancea* and *Metschnikowia*. These results are in agreement with previous reports which identified *Hanseniaspora*, *Metschnikowia*, *Lachancea,* and *Candida* as the dominant non-*Saccharomyces* genus in musts and grapes from different winemaking regions located in France, Spain, Italy, and China, while *Pichia*, *Torulaspora*, *Debaryomyces*, *Zygosaccaromyces*, *Issatchenkia*, *Rhodotorula*, *Rhodosporidium,* and *Cryptococcus* genus were identified in a lower frequency [12,26,54,55,56]. Contrary to previous reports, the *Candida* genus, namely *C. zemplinina*, a frequent dominant non-*Saccharomyces* species, was rarely observed in the studied Port wine must samples. Variations observed in the high diversity of yeast species from different vineyard regions, namely in the species proportion, are to be expected [57].

The success of wine fermentations involves not only the growth of desirable non-*Saccharomyces* and *Saccharomyces* species but also requires the selective development of strains within species. This requires the characterization of non-*Saccharomyces* species at the strain level. RAPD-PCR analysis of non-*Saccharomyces* yeasts isolated from spontaneously fermenting Port wine musts showed high strain variability within species, higher for *H. uvarum* isolates, suggestive of no clonal dominance during AF. Indeed, high genetic polymorphism was previously described for *H. uvarum* strains [8,39,42].

### 4.2. Technological Characterization of Selected Non-Saccharomyces Species

During AF yeasts are simultaneously and sequentially exposed to various stressors, including osmotic pressure, acidic pH, temperature increase and, at the end, high ethanol concentration, which inhibits yeast growth and leads to the finalization of AF. As expected, phenotypic screening evidenced that strain growth decreased with ethanol in a concentration-dependent way. Higher ethanol tolerance is a requirement for an efficient AF and it is dependent on the species and strains, in favor of *S. cerevisiae* dominance towards non-*Saccharomyces* species [58]. In agreement with previous studies, *L. thermotolerans* and *I. orientalis* strains were less sensitive to ethanol effects whereas the growth of *H. uvarum* and *M. pulcherrima* strains was more affected. Indeed, *L. thermotolerans* strains have been shown to be tolerant to ~10% ethanol, *H. uvarum* to ~7%, and *M. pulcherrima* to ~4–5% [59].

Wine yeasts are exposed to high temperatures during AF [60]. Without controlled refrigeration must temperature could rise to more than 40 °C, compromising yeast viability and favoring the production of undesirable compounds [61]. The high growth diversity observed in the current study between strains at 37 °C is not surprising. At this temperature *M. pulcherrima* and *L. thermotolerans* strains were negatively affected, while *I. orientalis* demonstrated a growth advantage and *H. uvarum* strains presented a heterogeneous response, with some isolates demonstrating a marked growth advantage and others a disadvantage. Our results demonstrates a reduced tolerance to high temperatures for both *M. pulcherrima* and *L. thermotolerans* strains and are in line with previous reports [62,63]. Poorer tolerance to higher temperatures in *S. cerevisiae* [64] has been explained by changes in membrane lipid composition and the consequential effects in its permeability and integrity [65,66]. As expected, at 30 °C the growth of the isolates from all species was not negatively affected.

The growth performance of the selected strains was slightly affected by an acidic pH. Yeast capacity to proliferate at an acidic pH is important since adequate acidity of the grape juice is required to inhibit unwanted microbial contaminants during fermentation, particularly at the beginning, when the ethanol concentration is lower [67]. The osmotic stress induced by high sugar and sorbitol concentrations reduced the growth of the selected strains in a dose-dependent way. Replacement of glucose for fructose resulted in reduced growth for *L. thermotolerans* strains without affecting *H. uvarum* strains. This was expected since *L. thermotolerans* are known to have more difficulties in fermenting fructose than glucose, whilst *H. uvarum* has a marginal preference for fructose [68]. Culture medium containing a yeast nitrogen base supplemented with three essential amino acids (L-proline, L-glutamine, and L-arginine) resulted in severely reduced growth rates of the selected strains. These results are in line with previous reports, explained by the peculiar amino acid consumption requirements of non-*Saccharomyces* strains [69].

The ethanol production rate, sugar consumption, and metabolite quantification, including glycerol, mannitol, and organic acids (tartaric, malic, succinic, pyruvic, citric, acetic and lactic acids), also provide evidence of the high variability within the strains.

Ethanol production rates quantified for *I. orientalis*, all *L. thermotolerans*, *M. pulcherrima*, and some *H. uvarum* strains, are in agreement with previous reports. Indeed, for *I. orientalis* and *L. thermotolerans*, ethanol production rates of about 10% were reported, whilst lower ethanol production rates of about 4% were reported for *M. pulcherrima* and *H. uvarum* strains [59].

The selected *H. uvarum* strains showed a preference for fructose consumption and produced high levels of glycerol in our studies. The results obtained are in agreement with previous reports demonstrating that selected *H. uvarum* strains in mixed fermentation with *S. cerevisiae* increased the production of primary metabolites, such as glycerol and acetaldehyde, as well as the secondary metabolites, including terpenes, C13-norisoprenoids, acetate esters, ethyl esters, and fatty acids [22,23]. In agreement with previous studies [11], *M. pulcherrima* strains exhibited a preference for glucose and produced high levels of glycerol (6–8 g/L). *L. thermotolerans* strains also showed a preference for glucose consumption but produced moderate levels of glycerol (4–5 g/L).

The concentrations of tartaric and malic acids maintained similar profile trends for all strains, whilst levels of succinic, acetic, lactic, and citric acids, determined during the stationary phase (72 h), distinguished selected strains. Pyruvic acid showed a production/consumption kinetics tendency for all the selected strains, increasing during the yeast exponential phase (until ~20 h), and decreasing slowly during the yeast stationary phase, in agreement with a previous report [70].

*I. orientalis* and half of the *L. thermotolerans* strains (B9, F7, and F8), produced low levels of acetic acid, while the other half of the *L. thermotolerans* strains did not produce this acid under the experimental conditions. Not all *M. pulcherrima* strains produced acetic acid, in agreement with previous studies [71,72]. Interestingly not all *H. uvarum* strains produced high levels of acetic acid, contrary to previous findings [59]. For example, acetic acid production was undetectable for H3 and H11 *H. uvarum* strains, highlighting the enological potential for these selected strains.

Interestingly, two of the *L. thermotolerans* strains produced particularly high levels of lactic acid, a reported characteristic of this species [73]. The *I. orientalis* strain produced the highest levels of ethanol and glycerol, had the highest glucose consumption, and released the lowest concentration of organic acids. The results obtained are in good agreement with previous studies, demonstrating the accumulation of higher levels of these alcohols in the final wines; however, significant differences for organic acids concentrations were reported [16,59].

## 5. Conclusions

To our knowledge, this is the first study to identify and characterize non-*Saccharomyces* species and strain heterogeneity in isolates recovered from spontaneously fermenting Port wine musts. The three species *H. uvarum*, *L. thermotolerans,* and *M. pulcherrima* were the most representative non-*Saccharomyces* species accounting for ~89% of the isolates. Even if Port wine production does not require complete sugar metabolism, only producing around 4–7% alcohol prior to fortification, many of these non-*Saccharomyces* strains show production potential. The results presented herein suggest the potential of selected *L. thermotolerans*, *M. pulcherrima*, and *H. uvarum* strains for the possible development of new starters for natural modulation and optimization of Port wine vinifications and quality.

## Supplementary Materials

The following are available online at https://www.mdpi.com/2304-8158/9/2/120/s1, Figure S1: Representative image of DNA patterns of selected *M. pulcherrima* strains isolated from Port wine must samples, generated by RAPD-PCR analysis with the oligonucleotide M14. Figure S2: Cluster analysis of *H. uvarum* strains isolated from Port wine must samples fermented with Touriga Franca grape variety; Figure S3: Cluster analysis of *H. uvarum* strains isolated from Port wine must samples fermented with Touriga Nacional grape variety.; Figure S4: Cluster analysis of *L. thermotolerans* strains isolated from Port wine must samples.; Figure S5: Cluster analysis of *M. pulcherrima* strains isolated from Port wine must samples.; Figure S6: Heatmaps representing the growth performance of non-*Saccharomyces* strains isolated from Port wine must samples on stress conditions associated with alcoholic fermentation. Table S1: Details of must samples used in the study.; Table S2: Growth of selected non-Saccharomyces species isolated from Port wine must samples on stress conditions associated with alcoholic fermentation.; Table S3: Quantification of metabolite for selected non-Saccharomyces species isolated from Port wine must samples.
