# Supplementary material for: Identification and Characterization of Non-Saccharomyces Species Isolated from Port Wine Spontaneous Fermentations
Source: Foods. 2020 Jan 23;9(2):120. doi: 10.3390/foods9020120 (PMC7074312; doi:10.3390/foods9020120)
Supplement: Supplementary file 1 [file foods-09-00120-s001.pdf]

Supplementary Material

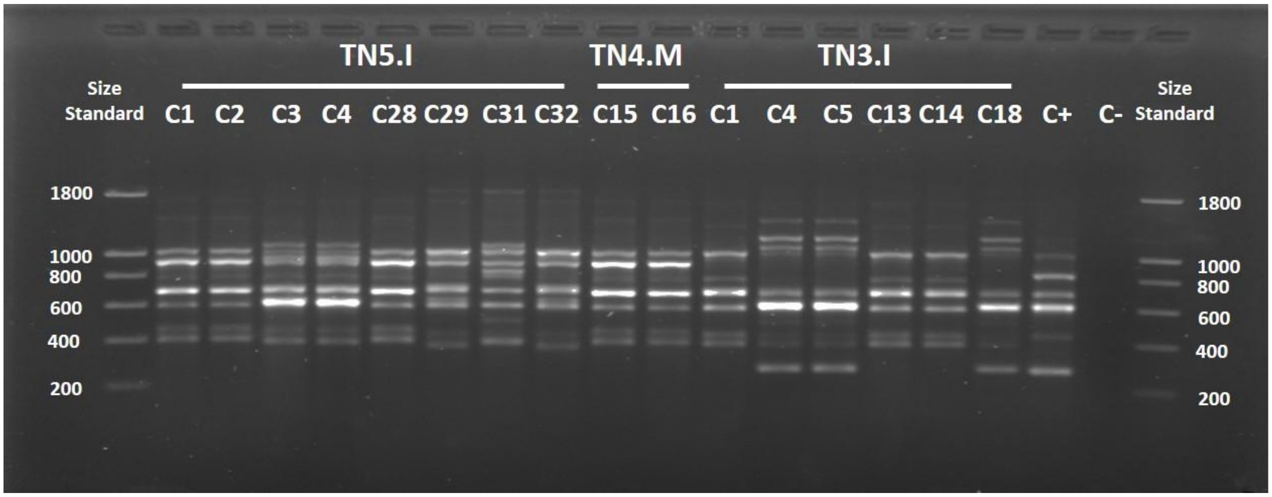

Figure S1: Representative image of DNA patterns of selected *M. pulcherrima* strains isolated from Port wine must samples, generated by RAPD-PCR analysis with the oligonucleotide M14. PCR products were analyzed on 1.5% agarose gel, in 1X Tris–borate EDTA, at 100 V for 2 h, and the size of DNA fragments was estimated by comparison with a DNA molecular weight marker (Size standard ranging from 200 to 1800 bp).

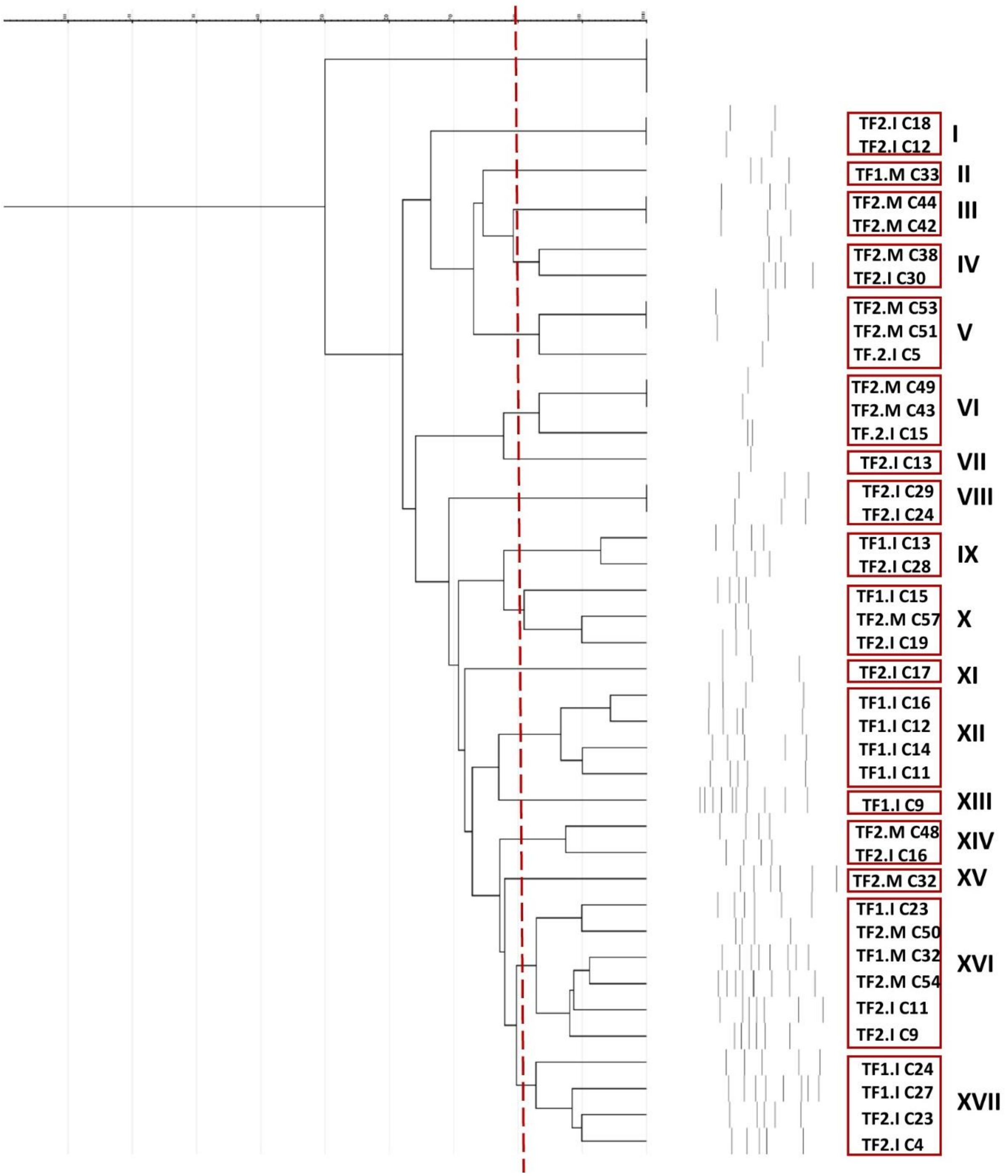

Figure S2: Cluster analysis of *H. uvarum* strains isolated from Port wine must samples fermented with Touriga Franca grape variety. Dendrograms were produced by hierarchical clustering of the electrophoretic patterns obtained by RAPD-PCR with the oligonucleotide P80. Dendrograms were generated using the Dice similarity coefficient with a tolerance of 10 and the UPGMA algorithm. Samples were considered of the same strain for similarity values ≥80% (red line). Groups of strains identified in roman numbers. Phylogenetic trees were constructed with GelJ v 2.0 software.

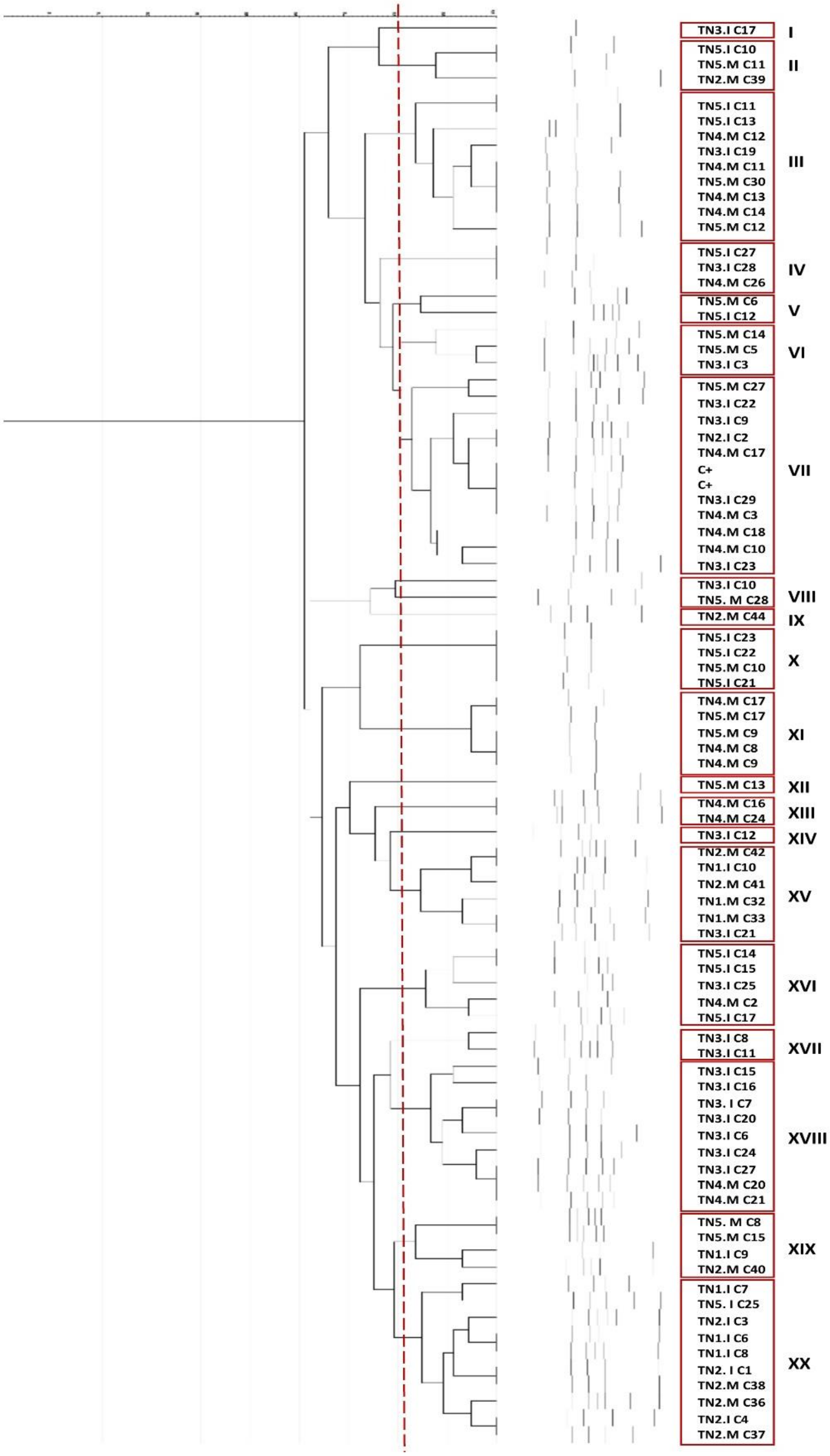

Figure S3: Cluster analysis of *H. uvarum* strains isolated from Port wine must samples fermented with Touriga Nacional grape variety. Dendrograms were produced by hierarchical clustering of the electrophoretic patterns obtained by RAPD-PCR with the oligonucleotide P80. Dendrograms were generated using the Dice similarity coefficient with a tolerance of 10 and the UPGMA algorithm. Samples were considered of the same strain for similarity values ≥80% (red line). Groups of strains identified in roman numbers. Phylogenetic trees were constructed with GelJ v 2.0 software.

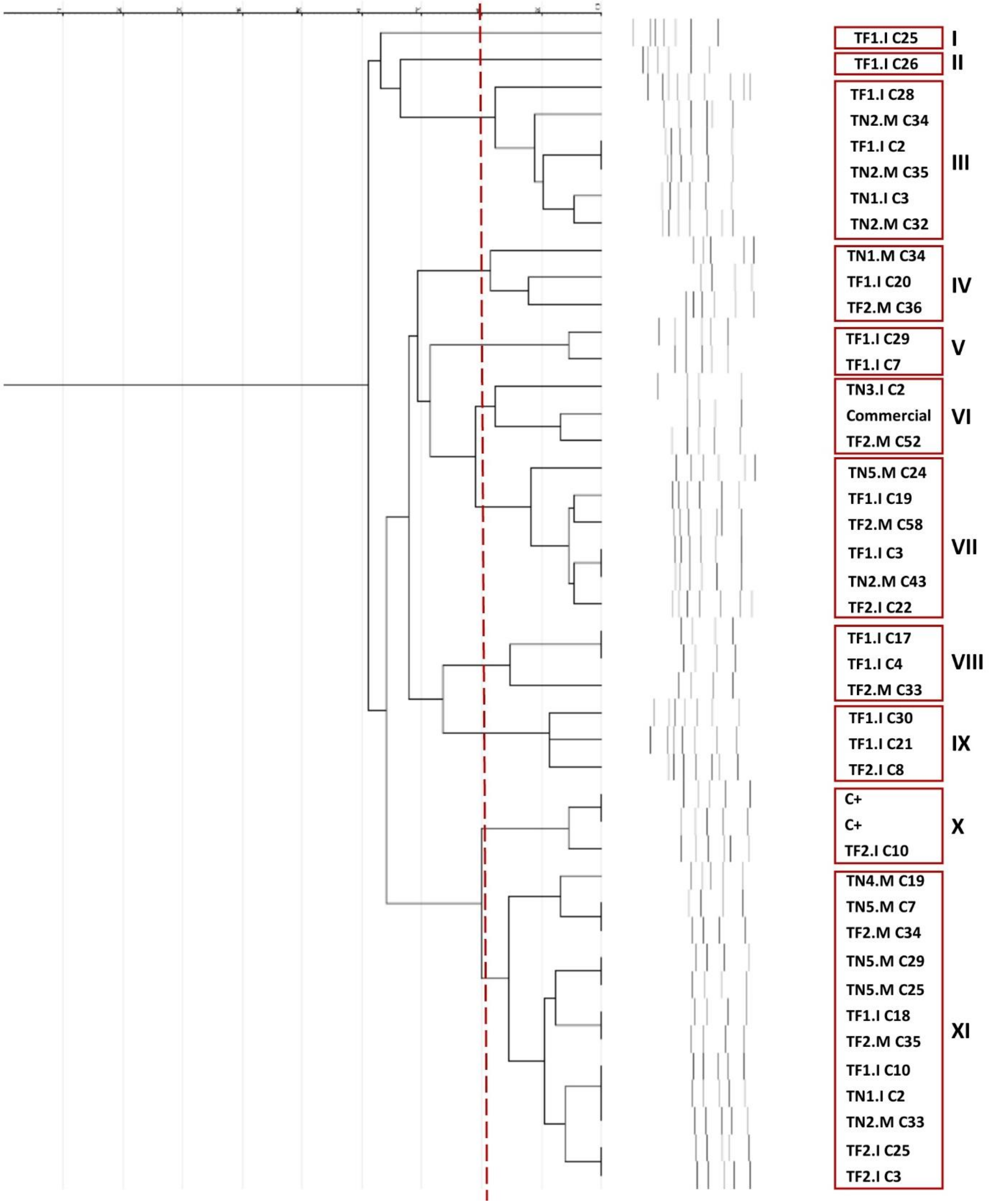

Figure S4: Cluster analysis of *L. thermotolerans* strains isolated from Port wine must samples. Dendrograms were produced by hierarchical clustering of the electrophoretic patterns obtained by RAPD-PCR with the oligonucleotide M14. Dendrograms were generated using the Dice similarity coefficient with a tolerance of 10 and the UPGMA algorithm. Samples were considered of the same strain for similarity values ≥80% (red line). Groups of strains identified in roman numbers. Phylogenetic trees were constructed with GelJ v 2.0 software.

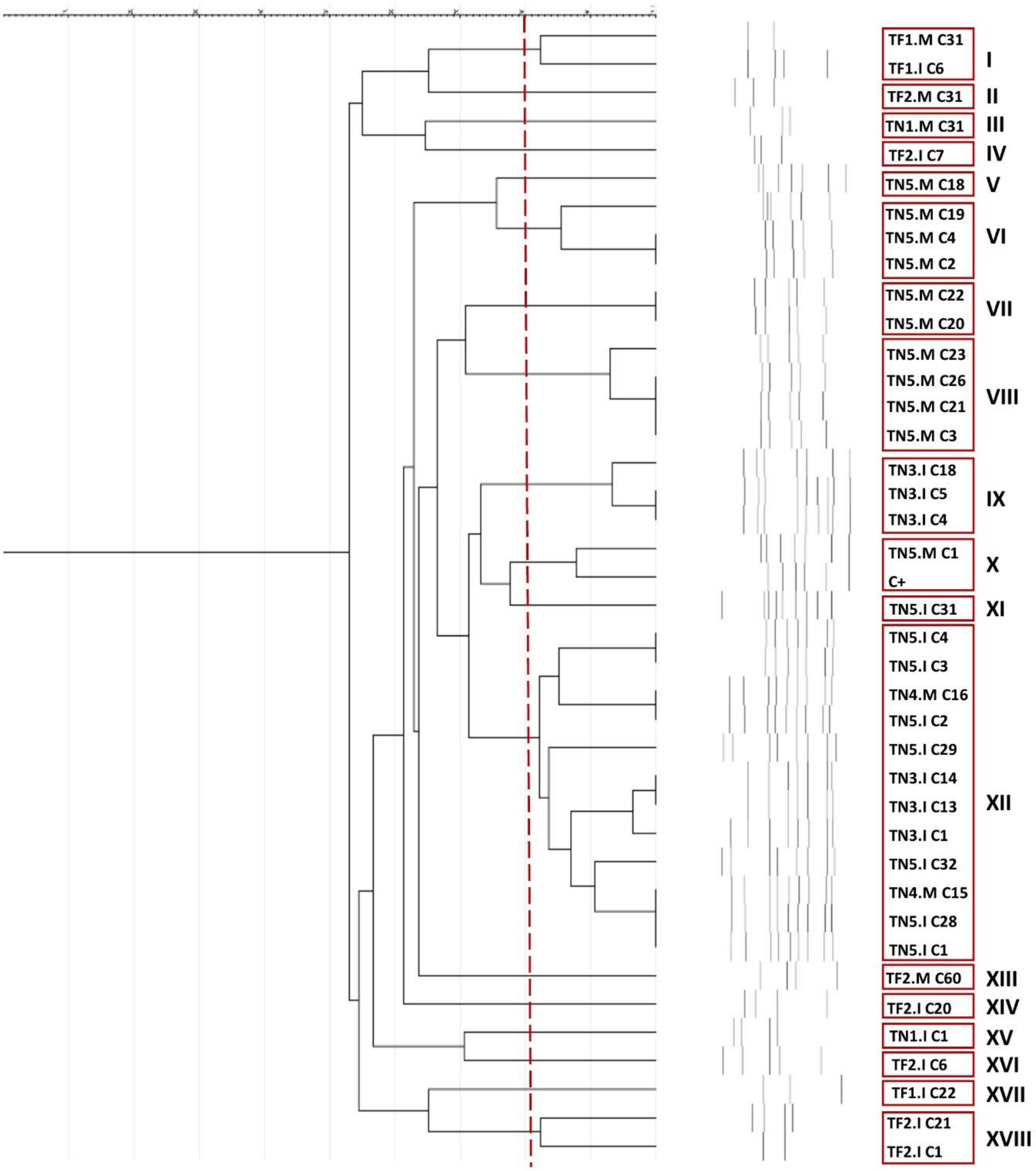

Figure S5: Cluster analysis of *M. pulcherrima* strains isolated from Port wine must samples. Dendrograms were produced by hierarchical clustering of the electrophoretic patterns obtained by RAPD-PCR with the oligonucleotide M14. Dendrograms were generated using the Dice similarity coefficient with a tolerance of 10 and the UPGMA algorithm. Samples were considered of the same strain for similarity values ≥80% (red line). Groups of strains identified in roman numbers. Phylogenetic trees were constructed with GelJ v 2.0 software.

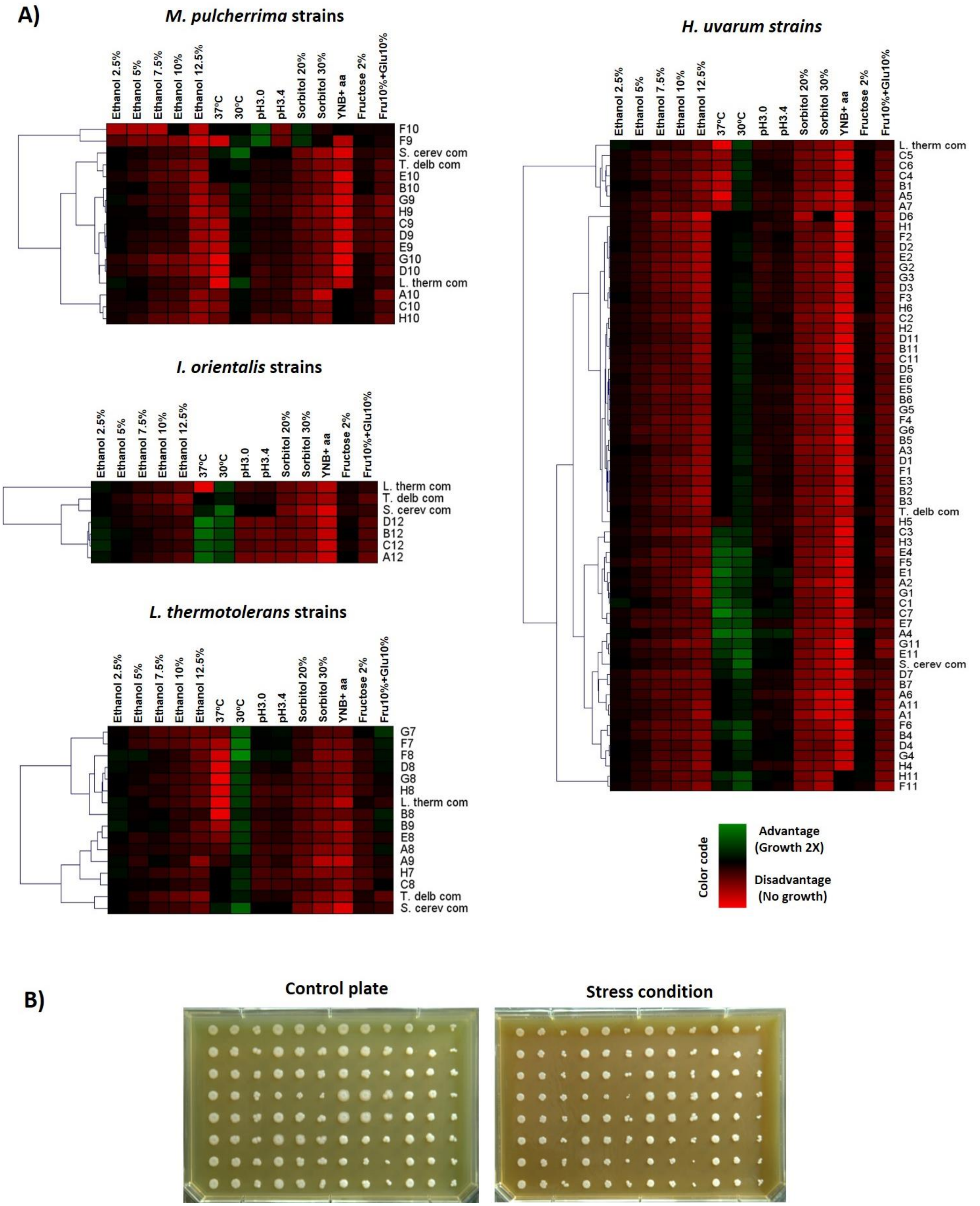

Figure S6: Heatmaps representing the growth performance of non-*Saccharomyces* strains isolated from Port wine must samples on stress conditions associated with alcoholic fermentation. A) Results represent the growth advantage (green) and disadvantage (red) of selected strains on YEPD culture plates

supplemented with stress conditions associated with alcoholic fermentation. Stress conditions include different concentrations of ethanol (EtOH), temperatures (37ºC and 30ºC), acidic pH (3.0 and 3.4), osmotic stress (Sorbitol 20%, and 30%), different nitrogen source (YNB + essential amino acids), and different carbon sources (Fructose 2% and Fructose 10% + Glucose 10%). For the assay were selected 92 non-*Saccharomyces* strains previously isolated, and as experimental controls two commercial non-*Saccharomyces*, respectively *L. thermotolerans*, and *T. delbrueckii*, and the commercial *S. cerevisiae*. Results represent the variation of the colony size area quantified in the plates supplemented with stress conditions, relatively to the quantified in the control plates (no stress). Results represent mean values of biological triplicates from three independent growth experiments. The hierarchical clustering of the strains was performed with Pearson correlation distance. B) Representative images of phenotypic screening growth plates of selected isolates. Isolates were grown in the absence (control plate) and in the presence of a selected stress condition (Ethanol 10%), during 48h.

Table S1: Details of must samples used in the study.

Must samples (N=22)	Wines selected for study (N=13)	Grape variety	Harvest year
TN1.I	TN1	Touriga Nacional	2016
TN1.M			
TN2.I	TN2		2016
TN2.M			
TN3.I	TN3		2013
TN4.I	TN4		2015
TN4.M			
TN5.I	TN5		2015
TN5.M			
TN6.M	TN6		2013
TN7.I	TN7		2015
TN7.M			
TN8.I	TN8		2016
TN8.M			
MIX.I	MIX	Touriga Nacional + Touriga Franca	2012
TF1.I	TF1	Touriga Franca	2016
TF1.M			
TF2.I	TF2		2015
TF2.M			
TF3.I	TF3		2015
TF3.M			
TF4.M	TF4		2016

Table S2: Growth of selected non-*Saccharomyces* species isolated from Port wine must samples on stress conditions associated with alcoholic fermentation.

sample	source	Strain	Ethanol 2.5%	Ethanol 5%	Ethanol 7.5%	Ethanol 10%	Ethanol 12.5%	37ºC	30ºC	pH 3.0	pH 3.4	Sorbitol 20%	Sorbitol 30%	YNB+aa	Fructose 2%	Fru10% + Glu10%
A1	isolated	2 H. uvarum	0,92948	0,891763	0,746751	0,655139	0,394511	0	1,136749	0,870275	0,922489	0,431123	0,179097	0,170929	0,856209	0,519523
B1	isolated	2 H. uvarum	1,007608	0,965205	0,779488	0,681629	0,429896	0,273553	1,165831	0,841661	0,904179	0,549555	0,485474	0,192849	0,948292	0,639785
C1	isolated	2 H. uvarum	1,135419	1,008323	0,870362	0,713341	0,506024	1,631694	1,360606	1,005285	1,10845	0,609054	0,515189	0,152436	0,941758	0,789435
D1	isolated	2 H. uvarum	0,943296	0,860659	0,681234	0,606867	0,430828	0	1,231137	0,858558	0,924405	0,574804	0,526624	0,189465	0,911789	0,648689
E1	isolated	2 H. uvarum	1,005562	0,884665	0,702598	0,627813	0,413701	1,694726	1,382061	1,075677	1,171153	0,5992	0,529961	0,172234	0,965148	0,837404
F1	isolated	2 H. uvarum	0,94772	0,840061	0,673186	0,557831	0,402743	0	1,099406	0,846696	0,911135	0,580484	0,550155	0,18744	0,970559	0,645555
G1	isolated	2 H. uvarum	1,005892	0,901772	0,730237	0,657085	0,417762	1,501868	1,201257	0,937345	1,01172	0,54961	0,494335	0,150564	0,913157	0,699715
H1	isolated	2 H. uvarum	0,941195	0,798042	0,620544	0,491784	0,341995	0	1,021701	0,797644	0,866328	0,564715	0,677607	0,083748	0,96921	0,578242
A2	isolated	2 H. uvarum	0,958259	0,841809	0,733226	0,608279	0,388265	1,544707	1,317452	0,96334	1,118539	0,489015	0,30996	0,124181	0,956937	0,729204
B2	isolated	2 H. uvarum	0,930795	0,842769	0,692878	0,595613	0,39006	0	1,155708	0,869084	0,933093	0,565149	0,515323	0,191326	0,986811	0,644262
C2	isolated	2 H. uvarum	0,925318	0,816379	0,649516	0,565371	0,38521	0	1,039921	0,870097	0,946054	0,509818	0,476035	0,111865	0,832245	0,574258
D2	isolated	2 H. uvarum	0,988289	0,792913	0,649454	0,56423	0,355323	0	1,146982	0,840755	0,941334	0,563	0,524162	0,143759	0,981437	0,657981
E2	isolated	2 H. uvarum	0,912275	0,749526	0,603563	0,507881	0,3423	0	1,12384	0,758902	0,882703	0,59053	0,529783	0,140754	0,932567	0,641442
F2	isolated	2 H. uvarum	0,945931	0,788194	0,61263	0,512345	0,33293	0	1,077189	0,879517	0,9793	0,620471	0,5576	0,16116	0,959053	0,695041
G2	isolated	2 H. uvarum	0,913828	0,755538	0,576752	0,490966	0,337929	0	1,014997	0,746826	0,859361	0,542299	0,458435	0,138011	0,948752	0,641273

sample	source	Strain	Ethanol 2.5%	Ethanol 5%	Ethanol 7.5%	Ethanol 10%	Ethanol 12.5%	37ºC	30ºC	pH 3.0	pH 3.4	Sorbitol 20%	Sorbitol 30%	YNB+aa	Fructose 2%	Fru10% + Glu10%
H2	isolated	2 H. uvarum	0,926648	0,853093	0,741492	0,645408	0,445318	0	1,144447	0,842179	0,906932	0,561103	0,504881	0,180452	0,928496	0,605576
A3	isolated	2 H. uvarum	0,994182	0,93473	0,768393	0,714855	0,456748	0	1,20265	0,849799	0,882167	0,510017	0,493864	0,240909	0,992064	0,643664
B3	isolated	2 H. uvarum	0,915451	0,805456	0,627619	0,57399	0,386719	0	1,173813	0,80057	0,888078	0,550228	0,497566	0,208422	0,992402	0,66708
C3	isolated	2 H. uvarum	0,919836	0,80534	0,624052	0,523059	0,44666	1,323483	1,282147	0,835384	0,918195	0,634801	0,536428	0,104022	1,00434	0,799218
D3	isolated	2 H. uvarum	0,956009	0,80183	0,625661	0,535377	0,372174	0	1,145375	0,774619	0,863413	0,59323	0,531773	0,178332	1,001153	0,618203
E3	isolated	2 H. uvarum	0,958846	0,869167	0,737485	0,657097	0,443695	0	1,201392	0,887657	0,937624	0,61661	0,575736	0,246872	1,004218	0,669959
F3	isolated	2 H. uvarum	1,013461	0,849131	0,708443	0,621237	0,420245	0	1,127588	0,810341	0,873855	0,619568	0,55659	0,212552	1,023842	0,669829
G3	isolated	2 H. uvarum	0,917651	0,782426	0,605412	0,542876	0,367908	0	1,062289	0,807071	0,882595	0,576264	0,512171	0,194516	1,028471	0,68376
H3	isolated	2 H. uvarum	0,957372	0,897626	0,818031	0,710559	0,61044	1,441972	1,21801	0,842011	0,958738	0,580364	0,523841	0,138995	1,024685	0,696566
A4	isolated	2 H. uvarum	1,039792	0,975464	0,813116	0,756651	0,546618	1,719673	1,652587	1,216383	1,254423	0,520951	0,455616	0,225585	1,045487	0,713337
B4	isolated	2 H. uvarum	0,945897	0,794234	0,643656	0,602588	0,459844	1,176955	1,515006	0,947583	0,991375	0,554202	0,491097	0,200041	0,989816	0,634247
C4	isolated	2 H. uvarum	0,930288	0,823654	0,640072	0,641231	0,436354	0,235747	1,288473	0,913455	0,938377	0,581222	0,582331	0,249281	0,982777	0,605117
D4	isolated	2 H. uvarum	0,995857	0,922541	0,717293	0,728522	0,531623	0	1,248497	0,995069	1,025923	0,587508	0,618777	0,252464	1,027066	0,638092
E4	isolated	2 H. uvarum	0,971903	0,838787	0,636874	0,651029	0,478096	1,591193	1,514371	0,943214	0,998756	0,548358	0,487532	0,199015	1,017026	0,592289
F4	isolated	2 H. uvarum	0,92083	0,782768	0,619086	0,614173	0,468176	0	1,172448	0,881007	0,89427	0,582042	0,514194	0,206389	1,052131	0,622508
G4	isolated	2 H. uvarum	0,999692	0,900209	0,685138	0,690886	0,486381	0	1,223026	0,982207	1,025593	0,589565	0,551857	0,231603	1,021036	0,599963

sample	source	Strain	Ethanol 2.5%	Ethanol 5%	Ethanol 7.5%	Ethanol 10%	Ethanol 12.5%	37ºC	30ºC	pH 3.0	pH 3.4	Sorbitol 20%	Sorbitol 30%	YNB+aa	Fructose 2%	Fru10% + Glu10%
H4	isolated	2 H. uvarum	0,966163	0,853167	0,633839	0,653607	0,430563	0	1,094238	0,831285	0,856281	0,510667	0,490872	0,209403	1,04009	0,55278
A5	isolated	2 H. uvarum	0,95167	0,809216	0,640991	0,71804	0,505821	0,077099	1,170834	0,772525	0,835589	0,508067	0,332446	0,138997	0,964874	0,640373
B5	isolated	2 H. uvarum	0,9434	0,797875	0,671917	0,647786	0,462568	0	1,12673	0,851678	0,860636	0,487951	0,42978	0,121029	0,954431	0,60954
C5	isolated	2 H. uvarum	0,925728	0,811468	0,654763	0,661192	0,495818	0,427986	1,202371	0,849185	0,841877	0,538246	0,487263	0,130072	1,000201	0,640124
D5	isolated	2 H. uvarum	0,89692	0,774229	0,652449	0,655248	0,517617	0	1,189129	0,950256	0,950847	0,557145	0,475756	0,150486	0,959351	0,714709
E5	isolated	2 H. uvarum	0,904126	0,750257	0,608278	0,604475	0,463261	0	1,17997	0,860267	0,839135	0,540739	0,44054	0,136967	0,971946	0,639003
F5	isolated	2 H. uvarum	0,8973	0,747044	0,632605	0,64388	0,56617	1,574973	1,439146	1,065129	0,999451	0,564056	0,429572	0,101159	1,021721	0,75431
G5	isolated	2 H. uvarum	0,900217	0,779087	0,679018	0,655272	0,520914	0	1,15775	0,853147	0,835732	0,524821	0,439047	0,162615	0,945483	0,580036
H5	isolated	2 H. uvarum	0,973199	0,833684	0,758799	0,714248	0,575593	0,777068	1,176163	0,881175	0,850688	0,515932	0,526931	0,170062	1,010236	0,609995
A6	isolated	2 H. uvarum	0,933009	0,790146	0,637243	0,678396	0,544497	0	1,181174	0,811449	0,798505	0,44313	0,210455	0,118821	0,965848	0,591211
B6	isolated	2 H. uvarum	0,926591	0,8068	0,67249	0,667626	0,556563	0	1,170983	0,905267	0,903442	0,520618	0,418675	0,113521	1,002602	0,632366
C6	isolated	2 H. uvarum	0,951614	0,806719	0,711691	0,755695	0,606937	0,473575	1,285746	0,939962	0,891599	0,582837	0,51514	0,17594	0,99819	0,663791
D6	isolated	2 H. uvarum	0,923678	0,825008	0,449445	0,486209	0,156132	0	1,020282	0,928767	0,862902	0,323336	0	0,073996	1,000471	0,524694
E6	isolated	2 H. uvarum	0,952943	0,804694	0,673147	0,698742	0,55804	0	1,249215	0,909318	0,907394	0,604474	0,506517	0,222033	1,012272	0,671167
F6	isolated	2 H. uvarum	0,974742	0,757401	0,623553	0,637443	0,546684	1,307728	1,431398	0,945552	0,893323	0,546335	0,438672	0,115504	1,052228	0,653166
G6	isolated	2 H. uvarum	0,92239	0,707092	0,65113	0,63709	0,460263	0	1,183111	0,890071	0,857565	0,590747	0,498514	0,158666	1,02169	0,611082

sample	source	Strain	Ethanol 2.5%	Ethanol 5%	Ethanol 7.5%	Ethanol 10%	Ethanol 12.5%	37ºC	30ºC	pH 3.0	pH 3.4	Sorbitol 20%	Sorbitol 30%	YNB+aa	Fructose 2%	Fru10% + Glu10%
H6	isolated	2 H. uvarum	0,906363	0,811476	0,695555	0,659014	0,432141	0,970833	1,106244	0,877186	0,850943	0,562849	0,444601	0,1551	1,000489	0,655523
A7	isolated	2 H. uvarum	0,900608	0,758741	0,686366	0,653322	0,567336	0,369181	1,215049	0,82424	0,812819	0,492271	0,397323	0,223717	0,717582	0,623585
B7	isolated	2 H. uvarum	0,9225	0,751534	0,681313	0,647008	0,571299	0	1,197767	0,926153	0,919428	0,531758	0,439193	0,224483	0,740726	0,63228
C7	isolated	2 H. uvarum	0,932135	0,795807	0,733851	0,680644	0,650949	1,819964	1,54496	1,135958	1,120832	0,629349	0,535708	0,231332	0,764547	0,805756
D7	isolated	2 H. uvarum	0,899826	0,704601	0,628949	0,560517	0,507598	1,121757	1,451887	0,907857	0,891465	0,527194	0,417486	0,153811	0,674889	0,682494
E7	isolated	2 H. uvarum	0,95723	0,744635	0,71143	0,632771	0,588942	1,473533	1,476071	0,901305	0,88655	0,533929	0,442107	0,181573	0,700613	0,596209
F7	isolated	3 L. thermotolerans	1,001103	0,78301	0,705746	0,662937	0,527342	0,339499	1,785508	1,012482	1,030862	0,784193	0,557926	0,552902	0,834856	1,16574
G7	isolated	3 L. thermotolerans	0,978401	0,832212	0,683812	0,657434	0,587121	0,60634	1,607409	1,023995	1,079851	0,835875	0,672245	0,651575	1,014904	1,323612
H7	isolated	3 L. thermotolerans	1,084901	0,920763	0,808939	0,721014	0,663419	0	1,266846	0,708683	0,742685	0,582611	0,484047	0,489412	0,709395	0,856317
A8	isolated	3 L. thermotolerans	0,944161	0,882772	0,885607	0,814404	0,767942	0,714962	1,382989	0,749084	0,761799	0,635385	0,488899	0,419823	0,770223	1,103072
B8	isolated	3 L. thermotolerans	1,084696	0,95694	0,985761	0,840179	0,83269	0,067274	1,218516	0,871651	0,891077	0,736596	0,613901	0,603198	0,775472	1,181258
C8	isolated	3 L. thermotolerans	0,998893	0,974935	0,955307	0,856457	0,767865	0	1,287114	0,720173	0,739072	0,75341	0,673826	0,47076	0,706643	1,009138
D8	isolated	3 L. thermotolerans	1,074603	0,955339	0,925772	0,803019	0,62541	0,105892	1,372908	0,911185	0,959008	0,677223	0,562392	0,520431	0,832845	1,080519
E8	isolated	3 L. thermotolerans	1,009117	0,832837	0,916224	0,771778	0,663048	0,544004	1,375927	0,810438	0,839518	0,684137	0,591888	0,43825	0,768772	0,880207

sample	source	Strain	Ethanol 2.5%	Ethanol 5%	Ethanol 7.5%	Ethanol 10%	Ethanol 12.5%	37ºC	30ºC	pH 3.0	pH 3.4	Sorbitol 20%	Sorbitol 30%	YNB+aa	Fructose 2%	Fru10% + Glu10%
F8	isolated	3 L. thermotolerans	1,123449	1,134638	0,944176	0,84197	0,602419	0,172411	1,899926	1,132679	1,119795	0,821908	0,506968	0,494339	0,803617	1,106452
G8	isolated	3 L. thermotolerans	0,99969	0,952771	0,825945	0,770734	0,675191	0,070518	1,446635	0,818346	0,844272	0,71368	0,62184	0,514812	0,782575	0,959312
H8	isolated	3 L. thermotolerans	0,996234	0,872217	0,894834	0,75831	0,637754	0,133645	1,403801	0,723133	0,740835	0,674479	0,626219	0,478569	0,784855	0,915907
A9	isolated	3 L. thermotolerans	1,062667	0,8269	1,030222	0,870216	0,458669	0,798838	1,13743	0,734328	0,865201	0,593574	0,283561	0,236492	0,754462	0,893141
B9	isolated	3 L. thermotolerans	1,157994	1,008033	1,06398	0,873546	0,641618	0,363051	1,397676	0,757262	0,840288	0,645761	0,516887	0,349672	0,780259	1,108129
C9	isolated	4 M. pulcherrima	0,985927	0,95982	0,923376	0,829952	0,431812	0,319269	1,039433	0,892025	0,902883	0,633019	0,545487	0,182216	0,707701	0,688431
D9	isolated	4 M. pulcherrima	0,977065	0,883781	0,839715	0,81479	0,385433	0,303719	1,06471	0,854157	0,864317	0,67626	0,571265	0,20713	0,712886	0,656142
E9	isolated	4 M. pulcherrima	1,011384	0,863122	0,768392	0,767008	0,357229	0,265988	1,125379	0,867616	0,874292	0,702196	0,561998	0,122837	0,736251	0,647026
F9	isolated	4 M. pulcherrima	0,670933	0,511192	0,520553	0,459899	0,181139	0,182769	1,209844	1,612486	0,590564	1,315334	0,956321	0,30046	0,986793	0,893442
G9	isolated	4 M. pulcherrima	1,058535	0,82008	0,73729	0,715818	0,324687	0,540141	1,128166	0,78959	0,774349	0,614065	0,4255	0,14847	0,756566	0,532604
H9	isolated	4 M. pulcherrima	0,969207	0,971274	0,781107	0,74186	0,330773	0,440774	1,17278	0,810387	0,804437	0,588975	0,461364	0,138681	0,746078	0,544422
A10	isolated	4 M. pulcherrima	0,940892	0,984949	0,800803	0,767622	0,449446	0,588508	1,052767	0,837486	0,814497	0,584777	0,231255	0	0,951025	0,516684
B10	isolated	4 M. pulcherrima	0,970067	0,999206	0,748978	0,695888	0,399749	0,69551	1,201819	0,822203	0,83175	0,698272	0,574678	0,199525	0,979644	0,714466

sample	source	Strain	Ethanol 2.5%	Ethanol 5%	Ethanol 7.5%	Ethanol 10%	Ethanol 12.5%	37ºC	30ºC	pH 3.0	pH 3.4	Sorbitol 20%	Sorbitol 30%	YNB+aa	Fructose 2%	Fru10% + Glu10%
C10	isolated	4 M. pulcherrima	0,924889	0,90138	0,732318	0,70414	0,435587	0,493396	1,042911	0,844525	0,829314	0,65277	0,541589	0	0,916512	0,714041
D10	isolated	4 M. pulcherrima	0,886224	0,816279	0,643885	0,590772	0,380683	0,135457	0,979293	0,729439	0,815539	0,664401	0,571189	0,090789	0,924958	0,653473
E10	isolated	4 M. pulcherrima	0,921482	0,804957	0,661897	0,64047	0,40168	0	1,000307	0,88685	0,898619	0,669318	0,526127	0,101763	0,933497	0,691587
F10	isolated	4 M. pulcherrima	0,345291	0,328027	0,275785	0	0,283184	0	0	1,533133	0,536099	1,306276	0,84925	0	0,932464	0,942774
G10	isolated	4 M. pulcherrima	0,848297	0,735528	0,524095	0,533716	0,362686	0,083143	0,956908	0,796248	0,823875	0,675761	0,527613	0,08373	0,925363	0,702962
H10	isolated	4 M. pulcherrima	0,942199	0,824955	0,64141	0,610649	0,419101	0,585654	0,979451	0,698549	0,700904	0,698584	0,509262	0	0,95138	0,626434
A11	isolated	2 H. uvarum	0,910392	0,856222	0,749803	0,653605	0,445528	0	1,124754	0,961046	0,901059	0,528198	0,25874	0,128731	0,955441	0,562842
B11	isolated	2 H. uvarum	0,921998	0,794429	0,695078	0,659502	0,437769	0	1,201035	0,924909	0,888419	0,544622	0,392857	0,063777	0,950799	0,621957
C11	isolated	2 H. uvarum	0,893157	0,801646	0,728488	0,679613	0,458584	0	1,243962	0,947964	0,915731	0,600743	0,468813	0,132179	0,967815	0,592849
D11	isolated	2 H. uvarum	0,94664	0,864146	0,767517	0,711832	0,503178	0	1,214481	0,98124	0,949506	0,585453	0,458975	0,103374	0,944196	0,605616
E11	isolated	2 H. uvarum	0,980969	0,843779	0,781198	0,675215	0,58438	1,34059	1,633195	1,073576	1,033502	0,529795	0,395784	0,098047	0,947976	0,530012
F11	isolated	2 H. uvarum	0,949076	0,832251	0,704516	0,636231	0,47144	1,121888	1,466344	0,991084	0,929787	0,547213	0,353087	0	0,93291	0,412333
G11	isolated	2 H. uvarum	0,945664	0,77519	0,689394	0,512219	0,498211	1,389822	1,473048	1,055116	1,021738	0,597504	0,386064	0,041425	0,971555	0,473933
H11	isolated	2 H. uvarum	1,013707	0,840944	0,757618	0,597418	0,587449	1,286289	1,491601	1,055889	0,997381	0,551908	0,377647	0	1,03648	0,514321

sample	source	Strain	Ethanol 2.5%	Ethanol 5%	Ethanol 7.5%	Ethanol 10%	Ethanol 12.5%	37ºC	30ºC	pH 3.0	pH 3.4	Sorbitol 20%	Sorbitol 30%	YNB+aa	Fructose 2%	Fru10% + Glu10%
A12	isolated	1 I. orientalis	1,120514	1,003743	0,88132	0,780771	0,686274	1,650321	1,389837	0,567591	0,529979	0,527748	0,426584	0,214241	0,994014	0,59514
B12	isolated	1 I. orientalis	1,208486	1,0592	0,939768	0,810642	0,713343	1,697947	1,448439	0,643966	0,577649	0,630709	0,542986	0,238663	1,013653	0,677369
C12	isolated	1 I. orientalis	1,240114	1,015359	0,914142	0,836415	0,785504	1,556391	1,451616	0,686337	0,618104	0,604849	0,553225	0,207667	0,926573	0,674979
D12	isolated	1 I. orientalis	1,147419	0,931808	0,851809	0,840649	0,782079	1,796309	1,523484	0,591703	0,572814	0,64656	0,527947	0,233161	0,957014	0,658464
SC com	commercial	6 S. cerevisiae	0,996669	0,934387	0,803285	0,766646	0,617416	1,173761	1,651601	1,001287	1,001953	0,567897	0,378874	0,136371	0,843832	0,792806
LT com	commercial	3 L. thermotolerans	1,104795	0,974523	0,841542	0,771818	0,629043	0,088773	1,388048	0,751676	0,816758	0,621837	0,503654	0,284514	0,932429	0,822507
TD com	commercial	5 T. delbrueckii	1,006617	0,862849	0,722482	0,600125	0,487608	0	1,153521	0,884628	0,897632	0,639346	0,496365	0,365552	0,893135	0,641966

Table S3: Quantification of metabolite for selected non-*Saccharomyces* species isolated from Port wine must samples.

Sample	Specie	OD	Glucose	Fructose	Ethanol	Glycerol	Mannitol	Citric acid	Tartaric acid	Malic acid	Acetic acid	Succinic acid	Lactic acid	Pyruvic acid
A2	*H. uvarum*	4,196333	33,83667	25,74	27,02333	6,076667	0,773333	0,2939	0,011467	-2,73757	0,456267	0,6753	0,1103	0,208333
A9	*L. thermotolerans*	3,657667	47,38	28,10333	26,71	4,983333	0,993333	0,0748	-0,2123	-3,04067	0	-0,61233	0,276467	0,0664
A10	*M. pulcherrima*	6,763333	34,90167	16,76333	20,72	4,745	10,15	1,274017	-0,5725	-3,71245	0	-0,1636	0,24925	0,074933
B8	*L. thermotolerans*	3,643	49,99333	35,35667	19,93333	5,163333	0,686667	0,3054	0,129867	-2,1441	0	-0,2872	3,459833	0,074
B9	*L. thermotolerans*	4,721	42,53667	27,06	23,14667	6,146667	1,31	0,279167	-0,1429	-2,7223	0	-0,12883	0,488467	0,0878
B10	*M. pulcherrima*	5,638667	75,39667	45,81833	31,11	6,585	15,675	2,425533	0,5015	-1,85783	0	1,868083	0,703383	0,171467
B12	*I. orientalis*	8,416667	87,57333	33,48	43,15	6,32	0,2	0,917267	-0,14223	-3,25693	0,2285	-0,1849	0,5677	0,082867
C1	*H. uvarum*	3,651333	35,57667	28,37333	19,48667	7,253333	1	0,325733	-0,11283	-2,95147	0,4959	2,6252	-0,0028	0,347567
C7	*H. uvarum*	3,595	17,37667	17,79667	16,92333	6,053333	0,93	0,1853	-0,14973	-2,4243	0,174167	1,296767	0,063267	0,2564
C8	*L. thermotolerans*	4,711	51,58333	34,70667	24,42	4,896667	1,466667	0,542133	0,224267	-2,4104	0	-0,5187	1,340767	0,0553
C9	*M. pulcherrima*	7,482	63,97	38,11	24,36	5,216667	16,10333	3,0421	0,14	-2,29473	0	0,461333	0,374367	0,117733
D3	*H. uvarum*	3,274667	8,82	12,40667	8,91	6,656667	0,633333	0,287067	0,1119	-1,74267	0,209967	0,653067	0,198133	0,223767
E1	*H. uvarum*	3,229667	23,71333	23,40833	9,435	5,366667	1,3	0,323567	0,000717	-2,24967	0	1,908833	0,02815	0,202067
E4	*H. uvarum*	2,996	17,30333	23,75667	11,21167	5,046667	0,38	0,28485	-0,09162	-2,57542	0,2383	0,0041	0,1554	0,229667

Sample	Specie	OD	Glucose	Fructose	Ethanol	Glycerol	Mannitol	Citric acid	Tartaric acid	Malic acid	Acetic acid	Succinic acid	Lactic acid	Pyruvic acid
E6	*H. uvarum*	2,996	50,5	45,14667	11,19667	7,14	2,096667	0,249533	0,0377	-2,44103	0,317367	1,114467	-0,06623	0,1785
E7	*H. uvarum*	3,486	14,76667	21,78	6,093333	3,143333	0,186667	0,270733	-0,27363	-2,1493	0	-0,1589	-0,08793	0,1458
E10	*M. pulcherrima*	9,084333	103,6533	78,54	31,18667	6,37	28,99667	6,200033	0,297867	-4,57903	0	1,046033	0,0809	0,167167
E11	*H. uvarum*	2,914333	0,143333	13,94	11,20667	5,583333	1,116667	0,043633	-0,87827	-4,41963	0	-0,13123	0,1809	0,1558
F5	*H. uvarum*	2,829	18,33	20,17667	8,296667	5,383333	1,436667	0,115167	-0,26637	-3,09393	0	1,5972	-0,0674	0,144267
F7	*L. thermotolerans*	5,125333	29,68333	18,41667	10,74	3,27	0,703333	0,456967	0,681533	1,787067	0	-0,77437	-0,06273	0,133
F6	*H. uvarum*	2,906667	5,29	11,87667	6,473333	3,273333	0,29	0,4859	0,242567	-0,4121	0	-0,1655	-0,10513	0,156233
F8	*L. thermotolerans*	5,325333	25,02667	15,55667	11,06667	3,616667	0,553333	0,460833	0,221633	-0,77397	0	-0,45793	-0,0001	0,122967
F11	*H. uvarum*	2,879	3,243333	14,18667	9,4	4,69	0,63	0,592633	-0,12367	-2,07423	0	0,387533	-0,01913	0,2386
G10	*M. pulcherrima*	9,525333	97,45	68,83	32,39333	4,856667	30,91667	4,593833	0,900467	-2,8693	0	1,4952	0,1901	0,1267
G11	*H. uvarum*	2,689333	12,01667	21,24333	10,25667	5,3	0,65	0,1073	-0,33963	-2,9938	0	0,0982	0,098233	0,223533
H2	*H. uvarum*	2,731333	24,63	22,31667	11,37	8,123333	2,63	0,578067	0,373	-2,21063	0,342767	1,735333	0,016867	0,182133
H3	*H. uvarum*	3,182667	22,99667	23,70333	5,643333	3,703333	0,74	0,301833	0,301067	-0,55923	0	1,887767	0,011933	0,180867
H6	*H. uvarum*	2,856667	30,04333	32,19667	6,4	5,413333	0,833333	0,306633	0,118133	-1,85827	0	0,323833	-0,02323	0,1219
H11	*H. uvarum*	2,554667	22,32	31,38333	7,59	4,796667	0,916667	1,002533	0,6594	0,0696	0	0,918967	-0,02823	0,323867
